# A Multi-Method Approach for Proteomic Network Inference in 11 Human Cancers

**DOI:** 10.1371/journal.pcbi.1004765

**Published:** 2016-02-29

**Authors:** Yasin Şenbabaoğlu, Selçuk Onur Sümer, Francisco Sánchez-Vega, Debra Bemis, Giovanni Ciriello, Nikolaus Schultz, Chris Sander

**Affiliations:** 1 Computational Biology Program, Memorial Sloan Kettering Cancer Center, New York, New York, United States of America; 2 Department of Epidemiology and Biostatistics, Memorial Sloan Kettering Cancer Center, New York, New York, United States of America; University of Zurich and Swiss Institute of Bioinformatics, SWITZERLAND

## Abstract

Protein expression and post-translational modification levels are tightly regulated in neoplastic cells to maintain cellular processes known as ‘cancer hallmarks’. The first Pan-Cancer initiative of The Cancer Genome Atlas (TCGA) Research Network has aggregated protein expression profiles for 3,467 patient samples from 11 tumor types using the antibody based reverse phase protein array (RPPA) technology. The resultant proteomic data can be utilized to computationally infer protein-protein interaction (PPI) networks and to study the commonalities and differences across tumor types. In this study, we compare the performance of 13 established network inference methods in their capacity to retrieve the curated Pathway Commons interactions from RPPA data. We observe that no single method has the best performance in all tumor types, but a group of six methods, including diverse techniques such as correlation, mutual information, and regression, consistently rank highly among the tested methods. We utilize the high performing methods to obtain a consensus network; and identify four robust and densely connected modules that reveal biological processes as well as suggest antibody–related technical biases. Mapping the consensus network interactions to Reactome gene lists confirms the pan-cancer importance of signal transduction pathways, innate and adaptive immune signaling, cell cycle, metabolism, and DNA repair; and also suggests several biological processes that may be specific to a subset of tumor types. Our results illustrate the utility of the RPPA platform as a tool to study proteomic networks in cancer.

## Author Summary

Pan-cancer proteomic datasets from The Cancer Genome Atlas provide a unique opportunity to study the functions of proteins in human cancers. Such datasets, where proteins are measured in different conditions and where correlations are informative, can enable the discovery of potentially causal protein-protein interactions, which may in turn shed light on the function of proteins. However, it has been shown that the dominant correlations in a system can be the result of parallel transitive (*i*.*e*. indirect) interactions. A wide suite of computational methods has been proposed in the literature for the discrimination between direct and transitive interactions. These methods have been extensively tested for their performance in gene regulatory network inference due to the prevalence of mRNA data. However, the understanding of the performance and limitations of these methods in retrieving curated pathway interactions is lacking. Here, we utilize a high-throughput proteomic dataset from The Cancer Genome Atlas to systematically test different families of network inference methods. We observe that most methods are able to achieve a similar level of performance provided their parameter space is sufficiently explored; but a group of six methods consistently rank highly among the tested methods. The protein-protein interactions inferred by the high-performing methods reveal the pathways that are shared by or specific to different cancer types.

## Introduction

The Cancer Genome Atlas (TCGA) Research Network has recently profiled and analyzed large numbers of human tumors both within and across tumor lineages to elucidate the landscape of cancer associated alterations at the DNA, RNA, protein, and epigenetic levels [1]. Integrated analyses of the resulting rich genetic and epigenetic data types have already started to shed light on commonalities, differences and emergent themes across tumor lineages [2,3]. Analysis of TCGA samples from 11 tumor types indicated that whole protein and phosphoprotein levels in these tumors, as measured by antibodies on reverse phase protein arrays (RPPA), capture information not available through analysis of DNA and RNA [4]. The RPPA platform used in the Akbani *et al*. analysis was subsequently expanded to include 187 high-quality antibodies, 51 of which are phosphospecific and 136 of which are non-phosphospecific. These antibodies were selected with a focus on cancer-related pathway and signaling events and analyzed with the intent to discover new therapeutic opportunities. This dataset is available for download from The Cancer Proteome Atlas [5], and referred to as PANCAN11 from here on.

### Analysis of function requires knowledge of interactions

The availability of proteomic datasets such as PANCAN11 where protein levels are measured across different conditions provides a unique opportunity to study the functions of proteins. However, the analysis of function requires knowledge of interactions. For instance, in the protein-folding domain, the function of a single residue during folding can be determined only by having knowledge about the residues it is interacting with. Similarly, the function of a protein in the cell can only be understood by determining its interaction partners. Therefore, the units of analysis are not the individual protein expression levels, but the interactions of proteins with other cellular entities.

Statistical techniques such as correlation can be used to study the interactions of proteins. However, correlation between two proteins does not imply that they **directly interact**, because correlation may also be induced by chaining of correlation between a set of intervening, directly interacting proteins. Such indirect correlations are called **transitive interactions**. It was previously shown that the dominant correlations in a system can be the result of parallel transitive interactions [6].

There are three main network motifs that lead to transitive interactions: fan-in, fan-out and cascade. A **fan-in** is a case where there are direct interactions from proteins A and B to a third protein C but there is no interaction between A and B. A **fan-out** is the situation where there is a direct interaction from protein C to both A and B but there is no interaction between A and B. A **cascade**, on the other hand, is a chain event where there are direct interactions from A to B, and from B to C, but not from A to C. In all these three cases, if the two direct interactions are in the same direction (both increasing or both decreasing), there is a transitive influence observed between the proteins that do not have a direct interaction. Since biological pathways and signaling events contain many fan-in, fan-out and cascade network motifs, transitive effects occur widely across the network and have previously been shown to be a systematic source of false positive errors for many computational network inference methods [7]. Thus, it is crucial to minimize transitive interactions when building network models from high-throughput datasets.

### A diverse array of computational network inference methods

A wide suite of computational methods has been proposed in the literature for the identification of direct interactions in networks. The common objective of many of these methods is to call a direct interaction between two entities if they are *‘not conditionally independent’* of each other given a set of other entities. One simple example is the regression-based **partial correlation** approach. Consider a three-variable system consisting of A, B, and C. When testing the existence of a direct interaction between A and B in this approach, measurements on A and B would first separately be regressed on the measurements on C, the residual vectors would be computed, and then the correlation between the residual vectors would be found. If this *‘partial’* correlation is significantly different from zero, a direct interaction is called between A and B.

Despite the similarity in the objective, these methods employ diverse inference procedures such as **mutual information** [8–11], **regression** [12–14], **Gaussian graphical models** [15,16], and **entropy maximization** [17,18]. The diversity of algorithms for inferring direct interactions, coupled with the absence of a robust off-the-shelf method, creates challenges for investigators that aim to generate hypotheses and eventually discover novel functional interactions among proteins. We address this challenge by testing different families of network inference methods towards the goal of deriving guidance for the better-performing methods.

### Evaluating the performance of network inference methods on a pan-cancer proteomic dataset

The RPPA platform, first introduced in Paweletz *et al*. [19] stands a good chance of becoming a widely used proteomics platform as greater numbers of reliable antibodies are being developed. Here, we present a rigorous comparison of the performance of 13 commonly used network inference algorithms based on PANCAN11, a pan-cancer RPPA dataset which contains levels of many proteins in a large number of samples, such that reasonably meaningful protein-protein correlations can be computed. The goal of this comparison is two-fold: To investigate 1) if the signal-to-noise ratio of the RPPA technology allows the discovery of known and novel protein-protein interactions (PPIs), and 2) to what extent algorithms that were originally developed for gene regulatory network inference accomplish the inference of PPIs.

Performance evaluation of PPI network inference for different cancers requires a ‘gold standard’ for each cancer type. However, a true gold standard for human PPIs does not exist, let alone a separate one for each tumor type. Most protein interactions in *in vivo* systems remain unknown or unproven and/or depend on physiological context. Yet public knowledgebases that store collections of curated pathway and/or interaction data contain useful information. For instance, **Pathway Commons** is a collection of publicly available and curated physical interactions and pathway data including biochemical reactions, complex assembly, transport and catalysis events [20], aggregated from primary sources such as Reactome, KEGG and HPRD and conveniently represented in the BioPAX pathway knowledge representation framework [21–24].

**Fig 1:**
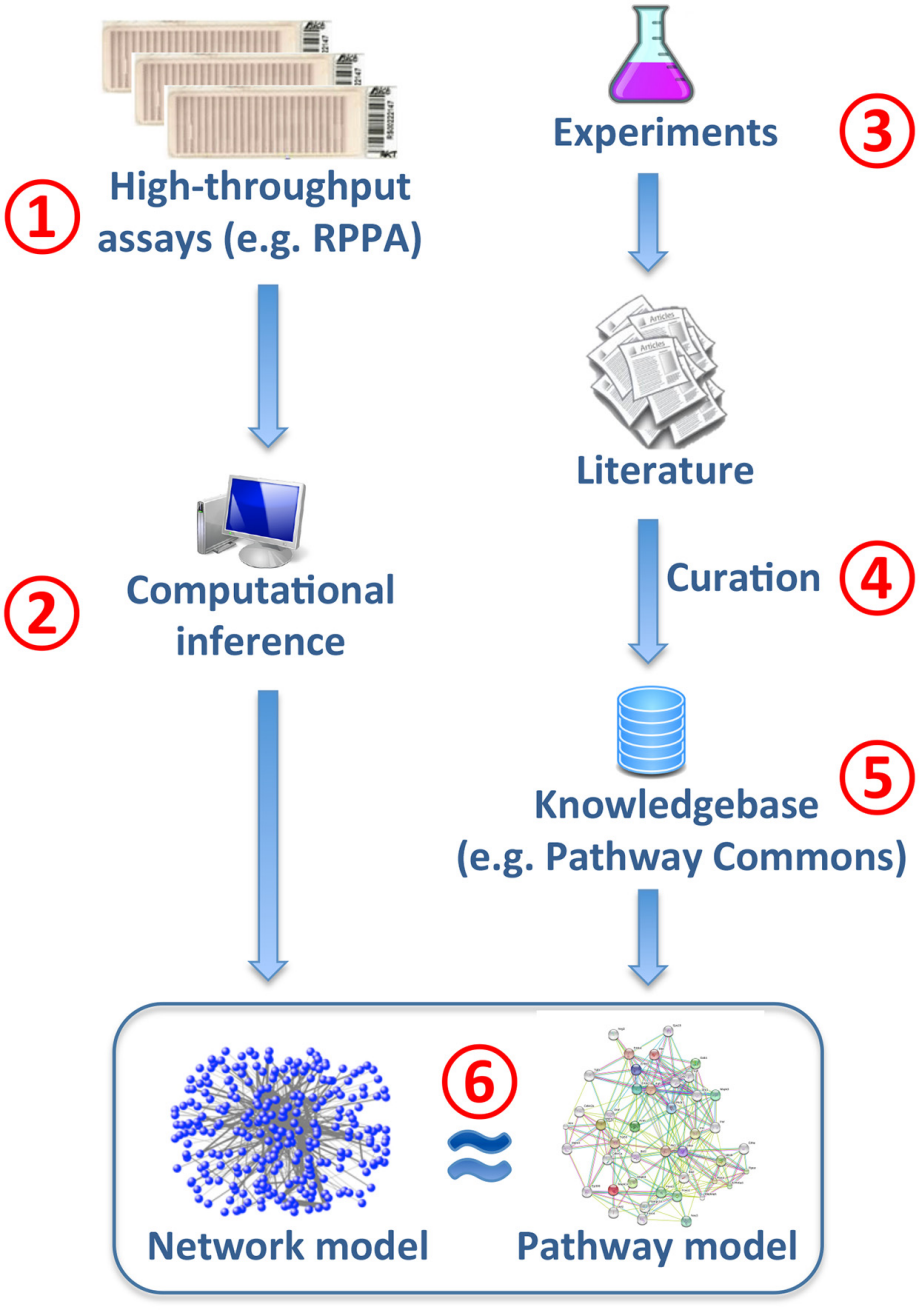
Workflow for the performance evaluation of network inference methods on a proteomic dataset. The workflow is comprised of a computational inference component and a pathway knowledgebase component that are used to generate separate PPI network models. Caveats involved in Steps 1–6 are discussed in Discussion and S1A Text.

In this study, we adopted Pathway Commons as a benchmark, and evaluated the performance of 13 network inference methods (Table 1) in their capacity to retrieve ‘true’ PPIs from RPPA datasets of 11 cancer types. We then used a group of high-performing methods to investigate the similarities and differences among the 11 cancer types in our dataset. The workflow of this study (Fig 1), involves the parallel generation of two PPI network models, one from *computational inference* and one from the *pathway knowledgebase*. On the inference side, multiple antibodies are assayed on an RPPA platform (Step 1) and the resulting dataset is normalized to generate a proteomic profile of the cohort such as PANCAN11. Computational network inference methods are then employed to create a network model with the inferred PPIs (Step 2). On the knowledgebase side, various wet-lab experiments are performed to generate data, and the resulting information is stored in the scientific literature (Step 3). Curators sift through the literature to distill multiple-layered information on PPIs (Step 4), and then this information is catalogued in knowledgebases such as Pathway Commons (Step 5). A comparison of the PPI network models from the two sides reveals the level of fidelity at which the ‘true’ network is constructed by the computational methods (Step 6).

**Table 1:** Tested network inference methods. Methods can be grouped according to the algorithm family or the regularization type. Algorithm families include correlation, partial correlation with inverse covariance, partial covariance with regression, and mutual information. Regularization types employed by methods can be shrinkage, sparsity, or a combination of shrinkage and sparsity as in ELASTICNET. The abbreviations used in this study are given in parentheses in the Method column.

Family	Method	Regularization	Running time* (sec)
Correlation	Pearson correlation (PEARSONCOR)	None	0.022
	Spearman correlation (SPEARMANCOR)	None	0.053
Partial correlation with inverse covariance	Simple partial correlation (SIMPLEPARCOR)	None	0.060
	GeneNet shrunken covariance matrix (GENENET)	Shrinkage	0.184
	Graphical lasso (GLASSO)	Sparsity	0.857
Partial correlation with regression	Partial least squares regression (PLSNET)	Shrinkage	109.370
	Ridge regression (RIDGENET)	Shrinkage	146.456
	Lasso regression (LASSONET)	Sparsity	2.99
	Elastic net regression (ELASTICNET)	Sparsity + shrinkage	6256.607
Mutual information	Algorithm for reconstruction of gene regulatory networks with additive penalty (ARACNE.A)	None	3.966
	Algorithm for reconstruction of gene regulatory networks with multiplicative penalty (ARACNE.M)	None	3.971
	Context-likelihood of relatedness (CLR)	None	3.995
	Network inference with maximum relevance / minimum redundancy feature selection (MRNET)	None	4.139

* Running time is for the optimal-parameter runs at limited recall, averaged over 11 tumor types.

The mutual information-based methods listed in Table 1 infer *unsigned undirected* edges, whereas the edges inferred by the ‘correlation’ and ‘partial correlation’ family of methods are *undirected* but *signed* (**positive** or **negative**). An undirected **positive** edge between A and B means that the direction of influence is not known, but A and B generally exist at both high or both low levels among all tested experimental conditions. A **negative** sign, thus, means that one is generally high when the other is low (tending towards mutual exclusivity). The use of positive/negative edges in this study refers to positive/negative signs of the weight. Also, we use the words ‘edge’ and ‘interaction’ interchangeably throughout the manuscript.

### Ascertainment bias in Pathway Commons

The workflow of performance evaluation as described above involves certain caveats. These are discussed in detail in the Discussion section and in S1A Text. Here we discuss one of the caveats, the *ascertainment bias* in pathway knowledgebases (Step 5 in Fig 1). Wet-lab experiments for PPI plausibly have over-representation of certain proteins due to the perceived interest in the field and ease of study. In a recent paper, a Pearson correlation of 0.77 was reported for the correlation between the number of publications in which a protein was mentioned and the number of interactions reported for that protein in literature-curated data [25]. This implies the potential existence of an ascertainment bias in pathway knowledgebases. More documented interactions of a certain protein will exist if that protein is studied more intensively by the community. The *ascertainment bias* in Pathway Commons precludes our benchmark network from being a perfect *gold standard*. This and other caveats challenge the comparability of pathway models from a knowledgebase and network models from a computational algorithm. Thus, it is necessary to be mindful of these caveats when interpreting the performance evaluation results in this study.

## Results

In this study, we evaluated the performance of 13 different network inference methods on the PANCAN11 RPPA dataset by using Pathway Commons as a benchmark. The PANCAN11 dataset is comprised of 3,467 samples and 187 antibodies. The total number of possible non-self interactions with 187 antibodies is 17,391. However, the number of interactions in Pathway Commons (version 2) involving any two antibodies from this set of 187 is 1,212 as determined by *PERA*[26] (Methods). This Pathway Commons benchmark subnetwork of 1,212 interactions forms the gold standard for this study, and has only 162 of the 187 antibodies as interaction partners (meaning interactions between the remaining 25 antibodies and any one of the full set of 187 antibodies are not included in Pathway Commons). As 162 antibodies can form a total of 13,041 non-self interactions, the gold standard network has a density of 9.29% (1212/13041).

### *Limited-recall* versus *full-recall* for the optimization of precision-recall curves

We obtained network predictions for 11 tumor types listed in Table 2 by using the 13 network inference methods listed in Table 1. We employed **precision–recall** curves to first find the optimal parameter values for each method, and then to compare the performance of methods using their optimal values. The precision-recall (PR) curves were constructed by first ranking an edge list based on significance, and then plotting precision and recall on the **y** and **x** axis respectively for cumulatively increasing numbers of the top (the most significant) edges from the list. The trade-off between precision and recall at different cutoffs gives a reliable idea about the performance of a method, and this performance can be quantified with the area under the precision-recall curve (AUPR).

**Table 2:** PANCAN11 tumor types, the abbreviations used in the study, and the number of samples in each tumor type.

Tumor type	Abbreviation	Number of samples
Bladder urothelial carcinoma	BLCA	127
Breast invasive carcinoma	BRCA	747
Colon adenocarcinoma	COAD	334
Glioblastoma multiforme	GBM	215
Head and neck squamous cell carcinoma	HNSC	212
Kidney renal clear cell carcinoma	KIRC	454
Lung adenocarcinoma	LUAD	237
Lung squamous cell carcinoma	LUSC	195
Ovarian serous cystadenocarcinoma	OV	412
Rectum adenocarcinoma	READ	130
Uterine corpus endometrioid carcinoma	UCEC	404
**Total**	PANCAN11	3467

The performance comparison for 13 methods was done separately for each tumor type. For a given tumor type, our procedure involved two steps. In the first step, we aimed to put all methods on an equal footing by finding each method’s optimal parameter values. This was achieved by running each method multiple times with different parameter values obtained from a one- or two-dimensional grid, computing the AUPRs for the resulting gene lists, and then finding the parameter or parameter combination with the highest AUPR. The parameters of each method and the design of the grid search are listed in Table A in S1E Text. In the second step, the highest AUPR values from all methods were compared to determine the method with the best performance. This procedure was repeated for each one of the 11 tumor types. Therefore the best-performing method may be different for each one of the tumor types.

**Fig 2:**
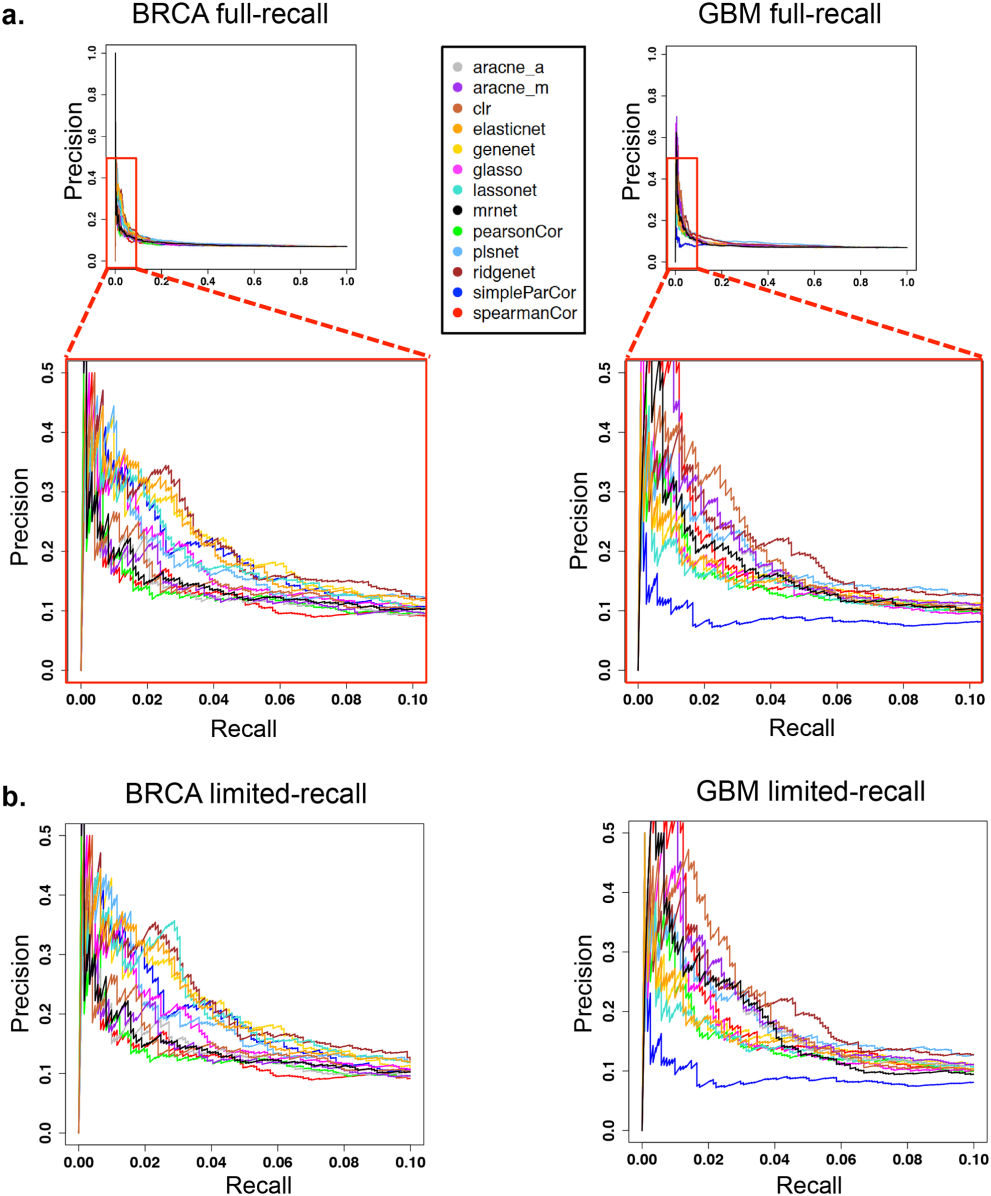
Precision–recall (PR) curves optimized for full versus limited range of recall values. (**A**) ***Top panel*:** PR curves for the 13 methods in the BRCA and GBM cohorts. PR curves are constructed by cumulatively increasing the number of edges from a ranked edge list. For each method, the relevant curve is computed with a choice of parameters that maximize AUPR in the recall range [0,1] (*i*.*e*. full-recall). ***Bottom panel*:** A zoomed-in version for recall in [0,0.1] and precision in [0,0.5]. (**B**) PR curves when the parameters are chosen to optimize AUPR specifically in the [0,0.1] recall range (*i*.*e*. limited-recall). We choose the limited-recall case for subsequent analysis because of two reasons. Beyond the 10% recall level, (1) the difference among methods become indiscernible, and (2) the precision level is very low suggesting network predictions are more likely to be affected by noise.

There is, however, a caveat concerning the computation of AUPRs from the entire span of the PR curves. We observe in PR curves that (1) there is no significant difference among methods beyond a 10% recall level, and (2) the precision level of network predictions is very low when recall is 10% or higher, suggesting that network predictions are more likely to be affected by noise. The PR curves for BRCA and GBM are shown in Fig 2A as representative examples of these two phenomena. Therefore, we chose to use AUPR only from the 0–10% recall range (i.e. **limited-recall**), and not from the entire recall range (i.e. **full-recall**) for the comparison of parameter configurations or the comparison of methods. As the parameter configuration that optimizes AUPR in the limited-recall span can be different from that in the full-recall span, some methods were observed to have different PR curves for the limited-recall case (Fig 2B). The subsequent analysis is carried out with network predictions from the limited-recall case. The optimal parameter values and the number of edges in the limited-recall case for each method and tumor type are shown in S5 and S6 Figs respectively.

### Performance comparison of network inference methods

**Fig 3:**
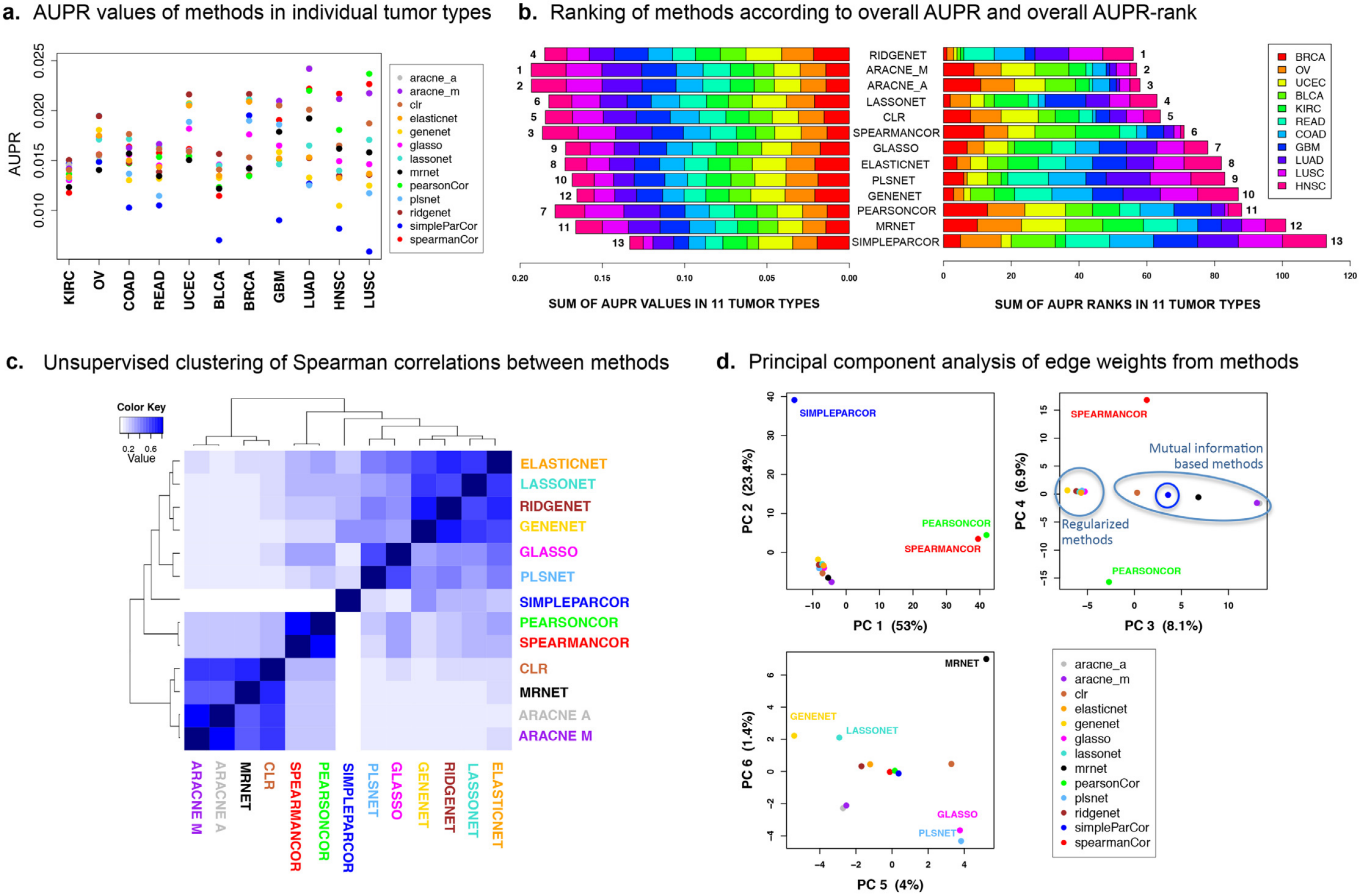
Performance comparison and unsupervised clustering for 13 network inference methods. (**A**) AUPR for each method in individual tumor types. Tumors are ordered according to increasing coefficient of variation. (**B**) Ranking of methods according to (left panel) overall AUPR and (right panel) overall AUPR rank in 11 tumor types. (**C**) Unsupervised hierarchical clustering of the Spearman correlations between methods. (**D**) Principal component analysis of edge weights from the methods by stacking edge lists from the investigated tumor types.

After identifying the PR curves to compare the methods, we asked whether any particular method is a clear winner by being the best in all of the 11 tumor types. The AUPR values in Fig 3A indicate that there is no single method that performs the best for all investigated tumor types. The tumor types in this figure are ordered from left to right according to increasing coefficient of variation. The differences in the tumor-wise AUPR means and variances indicate that the 11 tumor types are not equally amenable to network inference with RPPA data. These differences could partially be explained by the different statistics of inferred networks such as average-node-degree and network density, which we found to be negatively correlated with AUPR (Spearman r = –0.626 and –0.453 respectively) (S1B Text, S1 Fig).

Given the absence of a clear winner among the methods, we next asked what the overall best-performing methods were. To achieve an overall comparison of the methods, we ranked them across all tumor types based on (1) overall AUPR and (2) overall AUPR rank. For these two criteria, we computed respectively the sum of a method’s AUPR values in the investigated tumor types (Fig 3B, left panel), and the sum of its AUPR ranks in the same tumor types (Fig 3B, right panel). The different-colored segments in horizontal bars correspond to tumor types as shown in the legend. The numbers next to the horizontal bars indicate the rank of the method for the relevant criterion. Higher AUPR values but lower AUPR ranks indicate better performance. Therefore, the best rank of 1 is given to the highest overall AUPR and the lowest overall AUPR rank.

We observe in Fig 3B that the overall AUPR values (left) did not show as wide a variability across methods as the overall AUPR ranks (right). This might be due to the **overfitting** of the methods to the benchmark network, as each method was run with parameters that optimize performance (AUPR) against the same benchmark. The small differences in overall AUPR values suggest that these methods may have a general capacity to achieve similar performance in other contexts as long as their respective parameter space is sufficiently explored. However, such similarity in performance does not preclude the possibility that some methods consistently outperform others even if by small margins. To investigate this possibility, we ordered the methods from top to bottom according to increasing overall AUPR rank. This choice in the ordering shows that *RIDGENET* is the best-performing method overall. Broken down by tumor type, *RIDGENET* is the best for BRCA, OV, UCEC, BLCA and KIRC; but is not as good as *ARACNE* variants for HNSC, LUSC, LUAD, GBM, COAD, and READ. On the poor performance side, *SIMPLEPARCOR* has the worst rank according to both the overall AUPR and the overall AUPR rank (Fig 3B).

### Network predictions cluster methods primarily based on algorithm family

We next investigated the level of similarity among the network predictions of all 13 methods. One question here is whether the network predictions, as given by the inferred edge weights, would cluster the methods according to shared properties, such as the regularization technique, or the algorithm family listed in Table 1. To this end, we created one vector for each method by stacking the relevant edge weights from all 11 tumor types. We then computed the *Spearman correlation* between each pair of methods, and also performed dimensionality reduction on the same vectors using *principal component analysis* (PCA). Unsupervised clustering on the Spearman correlation matrix (hierarchical clustering with complete linkage and Euclidean distance) and PCA on the edge weight matrix reveal concordant results in terms of the grouping of the methods (Fig 3C and 3D). We observe three major groups of methods in Fig 3C: (1) Mutual information-based methods *ARACNE* (variants), *CLR*, *MRNET*, (2) correlation-based methods *SPEARMANCOR* and *PEARSONCOR*, and (3) partial correlation-based methods. *SIMPLEPARCOR* from the third group can be considered an outlier compared with the other partial correlation methods. Therefore, if we remove it as a separate group, the remaining partial correlation methods *RIDGENET*, *LASSONET*, *ELASTICNET*, *PLSNET*, *GLASSO*, *GENENET* can also be categorized as **‘regularized methods’**.

In the PCA plots, the 1^st^ principal component (PC) primarily separates the correlation-based methods *SPEARMANCOR* and *PEARSONCOR* from the others, accounting for 53% of the variance (Fig 3D). Correlation methods are fundamentally different from other investigated methods because they do not attempt to eliminate transitive edges in any way. This defect could predict poor performance for both *SPEARMANCOR* and *PEARSONCOR*. However, the superior overall performance of the rank-based *SPEARMANCOR* compared with the value-based *PEARSONCOR* and several regularized methods (Fig 3B) could be due to the ability of *SPEARMANCOR* to capture nonlinear relationships and/or its robustness against outliers.

The 2^nd^ PC (23.4% variance) separates *SIMPLEPARCOR*, a method that is based on Gaussian graphical models and that employs the sub-optimal pseudo-inverse technique when the covariance matrix is singular. Even when the covariance matrix is non-singular, the inversion of the covariance matrix without any regularization is known to introduce defects into the inference procedure unless the number of samples is at least twice the number of features [16]. As the cohort sizes in this study are less than twice the number of antibodies (2*187 = 374) for 7 of the 11 tumor types (Table 2), it is not surprising that *SIMPLEPARCOR* has poor performance in these tumor types, hence the poorest overall performance by a margin (Fig 3B). Indeed, we can observe that the tumor types where *SIMPLEPARCOR* achieves relatively better ranks are BRCA, OVCA, KIRC, and UCEC, the four tumor types that have cohort size greater than 374 (Fig 3A and 3B and Table 2).

The 3^rd^ PC (8.1% variance) achieves the separation of mutual information methods from regularized methods. Mutual information-based methods have the capability to model nonlinear relationships, but are not able to infer the direction of the relationship. These two fundamental differences may account for the clear separation of these methods from the others. Principal components can achieve a separation of regularization-based methods only at the 5^th^ and 6^th^ PC, which account for as little as 4% and 1.4% of the variance respectively (Fig 3D).

### TOP6: a group of high-performers instead of a *“best”* method

The modest differences between overall AUPR values in the left panel of Fig 3B, and also the lack of a consistently best-performing method in all tumor types are reasons to refrain from recommending one method as the best off-the-shelf method for PPI inference. Therefore, we propose a set of **high performers** by taking into consideration both the overall AUPR and the overall AUPR rank criteria. The methods that rank in the top six according to both of these criteria are the same six methods: *RIDGENET*, *ARACNE-M*, *ARACNE-A*, *LASSONET*, *CLR*, and *SPEARMANCOR* (Fig 3B). This set of high performers, referred to as **TOP6** from here on, includes representative methods from all algorithm families in Table 1 except for inverse covariance-based partial correlation methods. This may be indicative of inverse covariance being a poor framework to model PPIs in cancer especially if the cohort size is not several times as large as the number of proteins. In contrast, linear measures such as correlation and (ℓ_1_- or ℓ_2_- regularized) partial correlation, and also nonlinear measures such as mutual information are all represented in the set of high performers. Although ARACNE-M and ARACNE-A differ only in the form of the threshold (*i*.*e*. multiplicative or additive) used to remove the weakest edge in a triplet, the networks inferred by these methods are a function of the user-specified threshold values (S1F Text), and thus are not necessarily similar.

### Determining the *rank threshold* for the unsupervised clustering of tumor types

We next asked how the network predictions from the TOP6 methods cluster the 11 tumor types. However, similar to the reduction from 13 methods to the TOP6 methods, it was necessary to apply a significance threshold for edges before performing the clustering. P-values were not a viable option as significance scores because several methods did not return p-values. Even if p-values were obtained from all methods, it would not be possible to combine the p-values in this study in a statistically sound way because all methods used the same data, hence violating the independence requirement. Therefore, we resorted to an alternative method and used edge ranks as a nonparametric proxy for the importance of edges.

For a given tumor type, we computed (1) *consensus edge ranks* by taking the average of ranks from the TOP6 methods, and (2) *consensus edge weights* by taking the average of weights again from the TOP6 methods. The consensus ranks served as a nonparametric proxy for our importance levels, while the consensus weights were used in the clustering steps. Comparing consensus edge ranks obtained from the TOP6 methods with those obtained from all 13 methods (ALL13) showed that the TOP6 methods yielded slightly higher AUPR than ALL13 against the Pathway Commons gold standard (S3B Fig, S1C Text). This finding confirmed the use of TOP6 as a superior choice over ALL13.

The number of edges to use for the unsupervised clustering of tumor types was determined in the following way. For a certain threshold, we extracted all edges from a given tumor type that have a consensus edge rank smaller (more significant) than the threshold level. We then formed a matrix of edges by tumor types by combining extracted edges from all 11 tumor types. Next, we computed the PCs constructed as a linear combination of the tumor-type vectors, and inspected the behavior of the percentage of variance explained by the first three PCs as the rank threshold was varied from 25 to 2000. We observed that the sum of the variance percentages from the first three PCs exhibited an inflection point at rank 425, and thus determined 425 as the consensus rank threshold that determined significant and non-significant edges in each tumor type (S4b Fig, S1D Text).

### Unsupervised clustering of tumor types by edge weights recapitulate recently published gene and protein expression-based groups

Using the consensus rank threshold of 425, we investigated the natural groupings in the set of 11 tumor types when each tumor type was represented with the consensus edge weights obtained from the TOP6 methods. The number of edges in each tumor type that pass the consensus rank threshold is shown in Table B in S1E Text. The union set of these significant edges from the tested tumor types has 1008 edges. We refer to this union set as the ***discovery set***, and use it to perform PCA and hierarchical clustering of tumor types. The edges in the discovery set and the corresponding weights in the 11 tumor types are given in S1A Table. We note that, among the 187 antibodies in our dataset, all but STAT3_pY705 has at least one interaction in the discovery set (N = 186).

**Fig 4:**
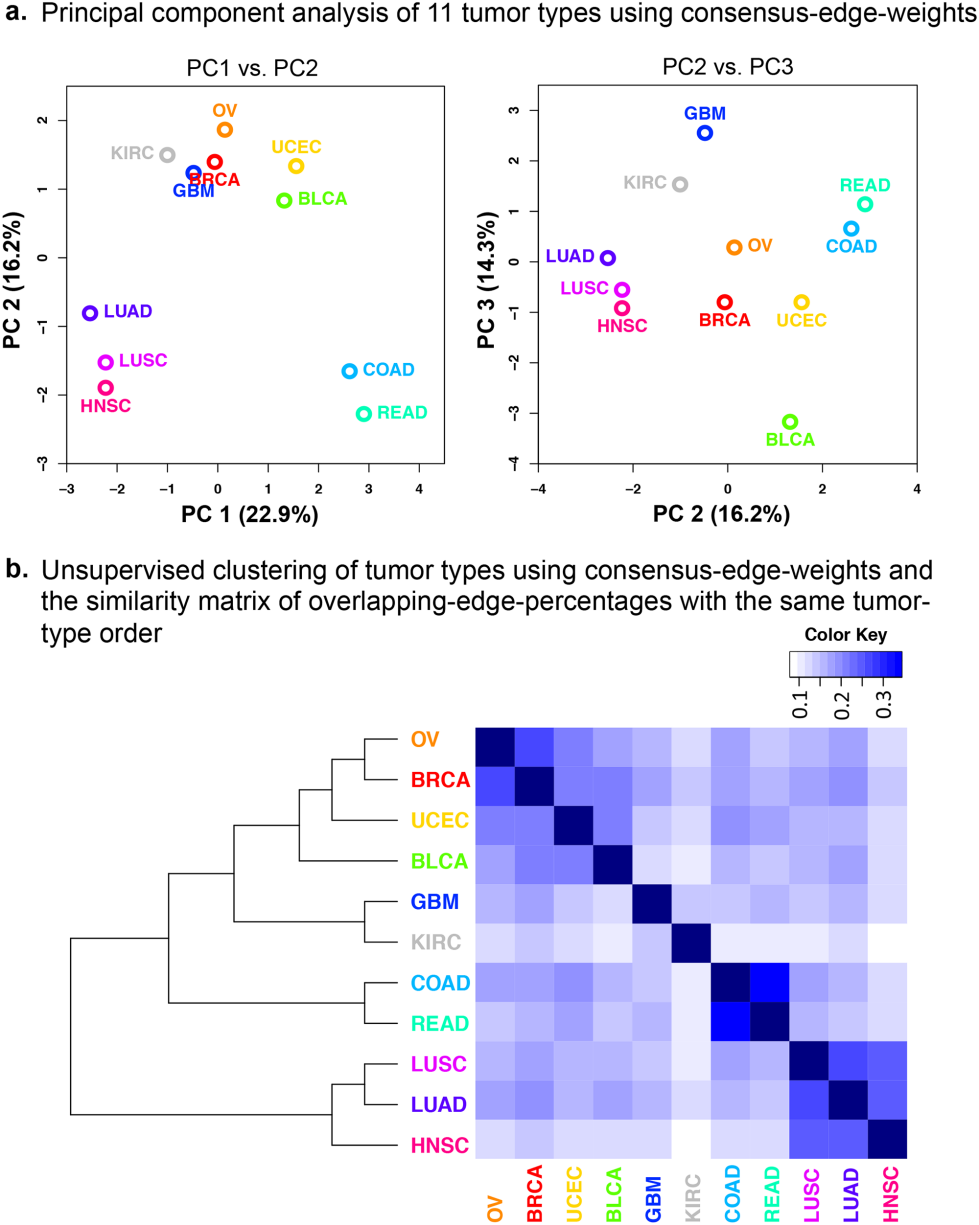
Principal component analysis and unsupervised clustering of 11 tumor types using consensus edge weights from the TOP6 methods. (**A**) Four major groups of tumor types can be observed in the PC1 vs. PC2 (left) and PC2 vs. PC3 (right) plots: 1) COAD, READ; 2) LUAD, LUSC, HNSC; 3) GBM, KIRC; 4) OV, BRCA, UCEC, BLCA. (**B**) Hierarchical clustering (Ward linkage and Euclidean distance) on consensus edge weights places tumor types into the same four groups on the dendrogram (left). The heat map on the right is constructed from the percentages of overlapping edges between tumor types. The order of tumor types in the heat map is taken from the dendrogram on the left.

We see in the PCA that PC1 and PC2 jointly separate the 11 tumor types into three groups, and also that PC3 further breaks down one group into two to result in a total of ***four groups***: 1) COAD, READ; 2) LUSC, LUAD, HNSC; 3) GBM, KIRC; and 4) OV, BRCA, BLCA, and UCEC (Fig 4A). These results are concordant with the clusters from hierarchical clustering (Fig 4B dendrogram) and also with the previously defined Pan-Cancer groups in the literature, as we elaborate below.

As for the first group, COAD and READ have previously been shown to cluster together in the Pan-Cancer subtypes defined both by RNA expression[27] and by protein expression[4]. These tumors have also been shown to have common DNA-based drivers (mutations and somatic copy number alterations), and hence have been treated as one disease [2,3,28]. Our finding that COAD and READ have the highest percentage of shared PPIs in this study (Fig 4B heat map) is also in line with these observations. Note that the order of tumor types in the heat map is taken from the dendrogram on the left, and that each cell represents the *Jaccard* index, *i*.*e*. the fraction of the intersection set over the union set of edges from two tumor types.

The tumors in the second group (LUSC, LUAD, and HNSC) have also been previously assigned to a single Pan-Cancer subtype in terms of protein expression[4]. However, RNA expression and somatic copy-number alteration (SCNA) data types have divided these tumor types into two groups: (1) a squamous-like subtype including HNSC and LUSC, and (2) a separate LUAD-enriched group [3,27]. In contrast to this separation where cell histology plays a more important role, both protein expression levels and PPI weights primarily separate these three tumor types based on tissue of origin: (1) lung-derived tumors LUAD and LUSC, and (2) a separate HNSC group (Fig 4B dendrogram and [4]).

Tumors in the third and fourth groups (GBM, KIRC, OV, UCEC, BRCA, and BLCA) can be separated along a continuum in the PC3 dimension (Fig 4A). However, we can consider GBM and KIRC as a separate group as these two tumor types separate from the other four in the unsupervised clustering dendrogram in Fig 4B. GBM and KIRC also cluster most closely among this set of 11 tumor types according to somatic copy-number alterations and protein expression levels [3,4]. However, KIRC also shows an outlier behavior for PPI networks in that it exhibits the lowest fraction of shared PPIs with other tumor types (Fig 4B). GBM, on the other hand, has an outlier property by being on one extreme of the separation along the PC3 dimension. This may reflect the fact that GBM samples arise from **glial cells** in the brain, a histological origin that shows marked differences from epithelial cells. Indeed, GBM was previously shown to have a distinct cluster comprised of only GBM samples in terms of both RNA and protein expression levels [4,27].

The fourth group contains OV, UCEC, BRCA, and BLCA; the first three of which are categorized as women’s cancers. The proximity of women’s cancers in clustering results may point to female hormones, such as estrogen and progesterone, causing a similar profile of PPI weights. BLCA is most similar to women’s cancers (Fig 4B), but it also is on one extreme of the separation along the PC3 dimension. This is concordant with the previously discovered Pan-Cancer subtypes because BLCA was shown to have the characteristic property of being one of the most diverse tumor types in the TCGA Pan-Cancer dataset. It had samples in 7 major RNA expression subtypes, and histologies in squamous, adenocarcinoma, and other variants in bladder carcinoma [27]. Next, we performed unsupervised clustering and community detection on the 1008 *discovery set* interactions to investigate patterns both among the interactions and also in the network formed by the interactions.

### Unsupervised methods reveal three groups of interactions and four densely connected modules in the discovery set network

**Fig 5:**
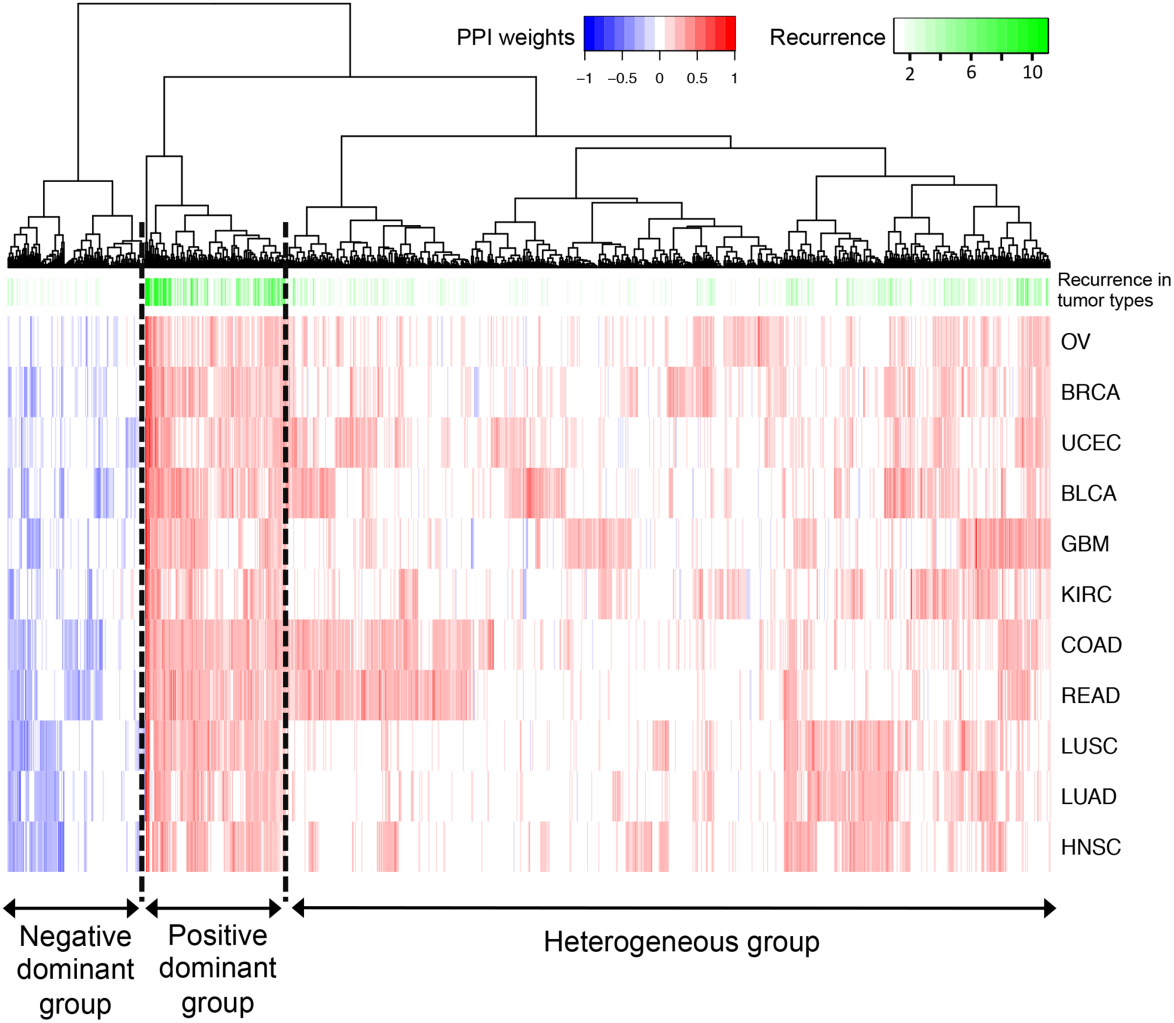
Hierarchical clustering of the 1008 edges in the *discovery set*. Three edge groups can be observed on the dendrogram: (1) positive dominant, (2) negative dominant and (3) heterogeneous. Hierarchical clustering was performed with Ward linkage and Euclidean distance. **Blue** and **red** denote negative and positive consensus edge weights respectively. The shades of **green** denote the recurrence of each edge, *i*.*e*. the number of tumor types (between 1 and 11) where that edge has consensus rank smaller (more significant) than the threshold of 425.

Unsupervised hierarchical clustering of the 1008 discovery set PPIs shows that these interactions form three main groups (Fig 5): (1) a **positive** dominant group where interactions generally have positive consensus weight and occurrence in multiple tumor types (mean pan-cancer weight = 0.25, mean pan-cancer recurrence = 4.42, N = 136, recurrence for an individual edge is computed over the binary values in S1B Table), (2) a **negative** dominant group where interactions generally have negative consensus weight (mean pan-cancer weight = –0.099, mean pan-cancer recurrence = 1.2, N = 133), and (3) a **heterogeneous** group (mean pan-cancer weight = 0.093, mean pan-cancer recurrence = 1.56, N = 739) which is a mixture of positive and negative, and also recurrent and non-recurrent interactions (S1A Table). In this set of 1008 most significant edges, both the number and the overall weight of negative interactions are smaller with respect to positive interactions. This may indicate either the lower prevalence of mutual exclusivity relationships for *in vivo* protein-protein interactions, or merely the difficulty of discovering negative PPIs from RPPA data.

**Fig 6:**
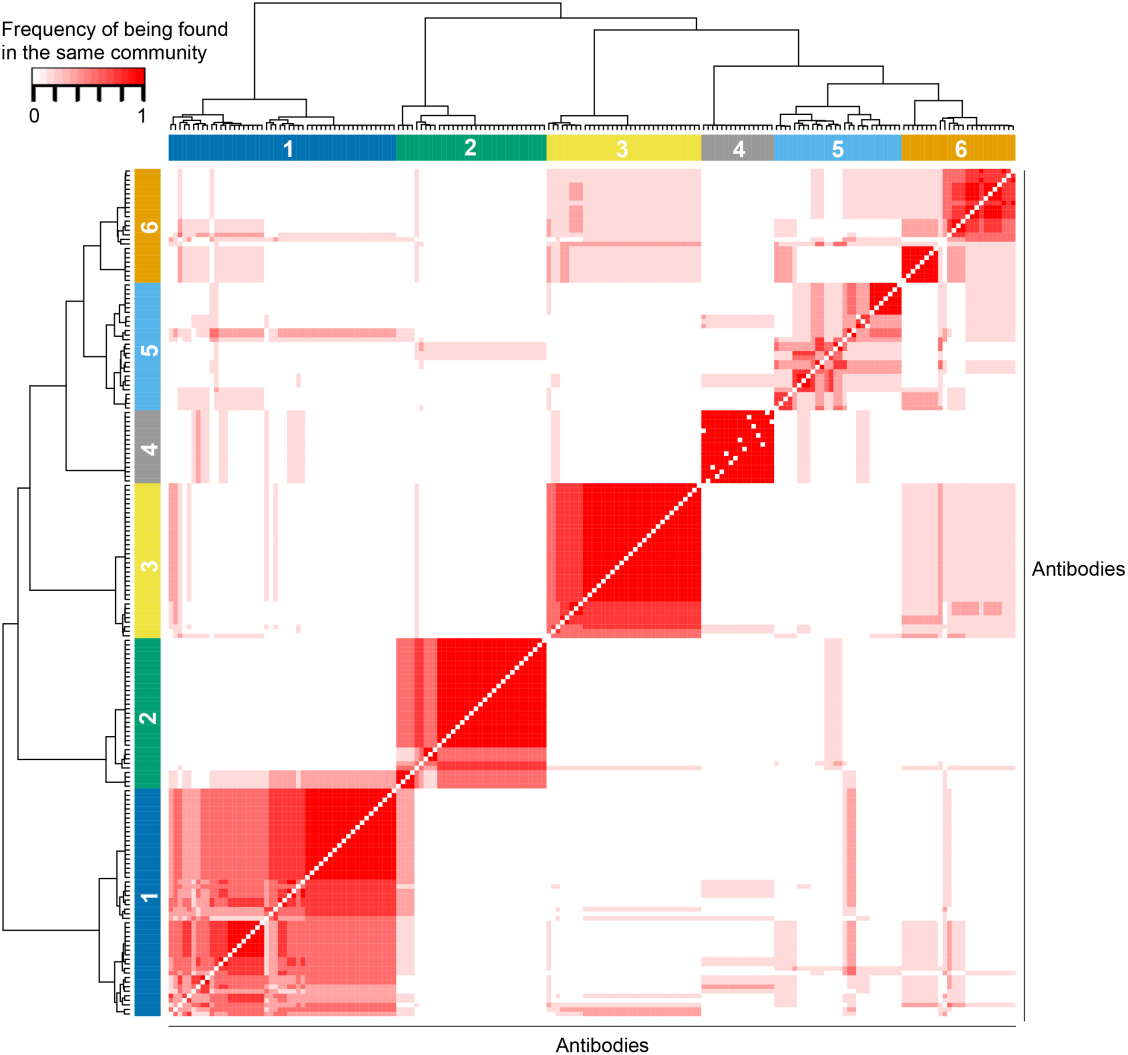
Consensus heatmap of predictions by community detection algorithms. Five algorithms were used to detect communities (modules) in the discovery set network formed by 186 antibodies. For each antibody pair, a frequency is computed to indicate the number of algorithms (out of five) that predict the pair to be in the same module. Hence, frequencies take values in {0,0.2,0.4,0.6,0.8,1}. The 186 x 186 matrix of frequencies is divided into six modules using Ward linkage hierarchical clustering with Euclidean distance. Modules 1–4 are robust, but Modules 5–6 are unstable or less robust.

We next visualized as networks the positive dominant, negative dominant and heterogeneous groups of interactions in order to gain insight on the related biological processes. However, the number of interactions in the heterogeneous group (N = 739) is too large to allow a clear interpretation of the results. Thus, we investigated whether the complete set of 1008 edges could further be broken down into densely connected **modules** (with high level of ***intra-module*** connectivity, and relatively lower levels of ***inter-module*** connectivity). To this end, we employed five different community detection algorithms: (1) fast greedy modularity optimization[29], (2) a spin-glass model from statistical mechanics coupled with simulated annealing for optimization[30], (3) multi-level modularity optimization[31], (4) an information theoretic approach that minimizes the expected description length of a random walker trajectory[32], and (5) random walk-based Walktrap community finding algorithm[33]. In the discovery set network, these algorithms detected 6, 8, 6, 11, and 22 modules respectively with similar and relatively high modularity scores (range 0.41–0.44, modularity due to [29]). Even though the number of detected modules was variable across the methods, we defined a consensus measure to identify the agreement/disagreement between the five predictions. For each antibody pair, the number of methods out of five, i.e. the frequency, of being predicted to be in the same module was utilized as a measure to quantify the level of method concordance. The consensus matrix of frequencies formed this way revealed four robust (Modules 1–4), and two less robust (Modules 5–6) modules among the discovery set interactions (Fig 6, S2A Table for consensus matrix, S2B Table for module membership of antibodies, S1A Table for module membership of interactions).

**Fig 7:**
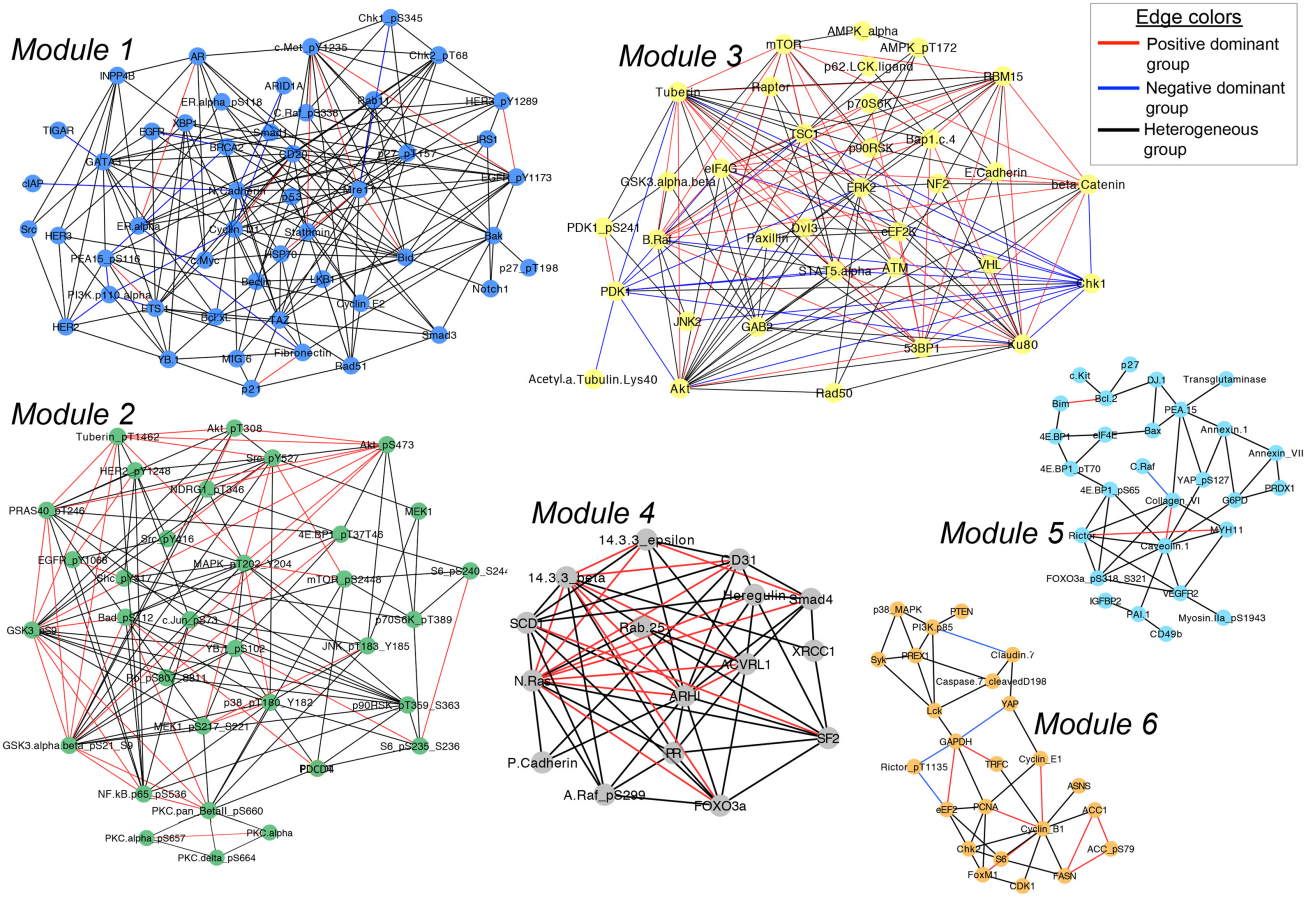
Network visualization of the six modules discovered in Fig 6. Only intra-module interactions are visualized. Each interaction is colored red, blue, or black based on membership in the positive dominant, negative dominant, or the heterogeneous groups respectively. The vertex colors are adopted from Fig 6 and denote module number. Network statistics are provided in Table 3, and module membership information for all discovery set interactions is provided in S1A Table.

The four robust modules (1–4) discovered in Fig 6 are also the densely connected ones with per antibody averages of 7.72, 8.24, 9.24, and 7.875 interactions respectively (Table 3). Mapping the positive dominant, negative dominant, and heterogeneous group memberships onto Module 1 reveals that 88% (170/193) of the edges in Module 1 are from the heterogeneous group (Fig 7). The major hubs in this module, N.Cadherin (22 edges), Mre11 (21 edges), and Bid (15 edges), have predominantly heterogeneous-group edges (Table 3). Interactions in this module may play roles in cell cycle, DNA damage repair, apoptosis, hormone and receptor tyrosine kinase (RTK) signaling pathways.

**Table 3:** Network statistics and major hubs in consensus modules visualized in Fig 7. Only intra-module interactions are considered (N = 640). The three consecutive numbers in parentheses in rows 1 and 4 indicate the number of edges in the ‘positive dominant’, ‘negative dominant, and the ‘heterogeneous’ groups respectively. The two consecutive numbers in parentheses in row 2 denote the number of non-phosphospecific and phosphospecific antibodies respectively. The sum of antibody counts in row 2 (185) is one less than the number of unique antibodies in the discovery set because one antibody (14.3.3_zeta) has no intra-module interactions despite having multiple inter-module interactions (S1A Table).

	MODULE					
ROW	1	2	3	4	5	6
**1. Number of edges**	193(11,12,170)	136(40,0,96)	157(44,21,92)	63(16,0,47)	46(3,1,42)	45(8,4,33)
**2. Number of antibodies**	50 (40,10)	33 (3,30)	34 (32,2)	16 (15,1)	28 (23,5)	24 (22,2)
**3. Average degree**	7.72	8.24	9.24	7.875	3.29	3.75
**4. Major positive or heterogen-eous group hubs**	N.Cadherin (22 total; 0,2,20)	GSK3.alpha.beta_pS21_S9 (16 total; 7,0,9)	Akt (20 total; 3,2,15)	N.Ras (14 total; 8,0,6)	Caveolin.1 (9 total; 1,0,8)	Cyclin_B1 (10 total; 3,0,7)
	Mre11 (21 total; 4,1,16)	GSK3_pS9 (16 total; 7,0,9)	Tuberin (19 total; 8,2,9)	ARHI (14 total; 3,0,11)	–	–
	Bid (15 total; 1,0,14)	MAPK_pT202_Y204 (16 total; 6,0,10)	Ku80 (18 total; 7,2,9)	–	–	–
**5. Major negative group hubs**	–	–	Chk1 (13 total; 0,12,1)	–	–	–
			PDK1 (11 total; 1,9,1)			

Modules 2 and 4 are distinguishable from the other modules by having zero edges from the negative dominant group, and an abundance of edges from both the positive dominant and heterogeneous groups (Table 3, Fig 7). Interestingly, 91% (30/33) of the antibodies in Module 2 are phosphospecific. On the contrary, only 6.2% (1/16) of the antibodies in Module 4 are phosphospecific. These results raise the question whether significant correlations can only be found between antibodies of the same type (*i*.*e*. phosphospecific with each other and non-phosphospecific with each other). To address this question, we analyzed the interactions in Module 1, which is the only module other than Module 2 that contains a subtantial number of phosphospecific antibodies (N = 10). In this module, phosphospecific antibodies have 64 interactions, but only 14 of these are between two phosphospecific antibodies (22%) (S3 Table). This result shows that it is possible to observe significant correlations between phosphospecific and non-phosphospecific antibodies, and suggests that there may be biological differences between Module 1 and Module 2 antibodies that lead to the differences in correlation patterns.

We next compared the biological functions of the phosphospecific antibodies in Module 1 and 2 to investigate potential differences. Processes such as cell cycle, proliferation, RTK signaling, RAS/MAPK signaling were shared between the modules; but Module 2 antibodies were involved in a greater variety of oncogenic pathways such as AKT/mTOR, Wnt, and NFκB signaling. Even though these differences do not rule out technical bias as a reason for the absence of non-phosphospecific antibodies in Module 2, they provide grounds for a biological reason such as the coordinated regulation of signal transduction pathways. An example of a technical bias that could lead an antibody to correlate highly only with another antibody of the same type would be that non-phosphospecific antibodies are expected to bind to both phosphorylated and non-phosphorylated states of a target protein, whereas phosphospecific antibodies only bind to target phosphosites (barring off-target activity).

Module 3 is the densest network among the six, with 9.24 interactions per antibody on average. This module almost exclusively contains non-phosphospecific antibodies (32 out of 34), but has a good representation of edges from all three of positive dominant, negative dominant and heterogeneous groups (Table 3, Fig 7). Akt, Tuberin, and Ku80 antibodies are major hubs (20, 19, and 18 edges respectively) with predominantly positive-dominant and heterogeneous-group edges. There is an absence of phosphospecific antibodies, but due to the cross-reactivity of non-phosphospecific antibodies, this module may be related to several signal transduction pathways (e.g. Akt, mTOR, B.Raf, β-catenin) and DNA double-strand break repair (e.g Ku80, Rad50). Interestingly, this module is the only one that has hubs with predominantly negative-dominant-group edges. Chk1 and PDK1 antibodies have, respectively, 12 negative-dominant-group edges out of 13 (92%), and 9 negative-dominant-group edges out of 11 (82%). One speculation for the underlying cause of the negative edges could be the mutual exclusivity relationship between processes that promote cellular proliferation and those that promote cell cycle arrest or apoptosis. Module 5 and 6 are relatively unstable communities with smaller numbers of intra-module interactions, some of which may play roles in cell cycle (CDK1, Cyclin_B1, Cyclin_E1), translation (eIF4E, 4E.BP1, 4E.BP1_pT70), and apoptosis (Bim, Bcl.2, Bax).

### Mapping *discovery* set edges to Reactome gene lists as a means of *interpretation*

The network visualization of the discovery set edges presents an opportunity to ‘*discover’* biologically interesting cancer-related interactions. However, a thorough understanding of the interactions in densely connected modules may still be challenging. To facilitate this *‘interpretation’* and potentially identify the biological processes that each interaction may take part in, we mapped the discovery set interactions to Reactome[21] gene lists. This mapping could not be performed with Pathway Commons because the only unit of analysis in Pathway Commons is interactions, *i*.*e*. Pathway Commons does not have previously defined gene lists as in Reactome.

The mapping of inferred interactions to Reactome gene lists involved multiple steps. We first obtained a complete set of Reactome gene lists (N = 1705), and filtered these to keep only the human-specific ones (N = 1669). We then reduced each inferred interaction to the genes corresponding to its interaction partners. If both antibodies corresponded to the same gene, the PPI was left out of the analysis as it would cause a self-interaction at the gene level. We then identified the Reactome gene lists that contained these two interacting genes, and increased their scores by the relevant consensus edge weight. Finally, gene list scores were normalized by the number of matching interactions and also the number of genes in the gene lists to obtain the tumor-type-specific ‘**average interaction strengths**’ (S4 Table) (Methods). 339 out of 1669 gene lists had a match with at least one of the discovery set interactions in one of the tested tumor types.

**Fig 8:**
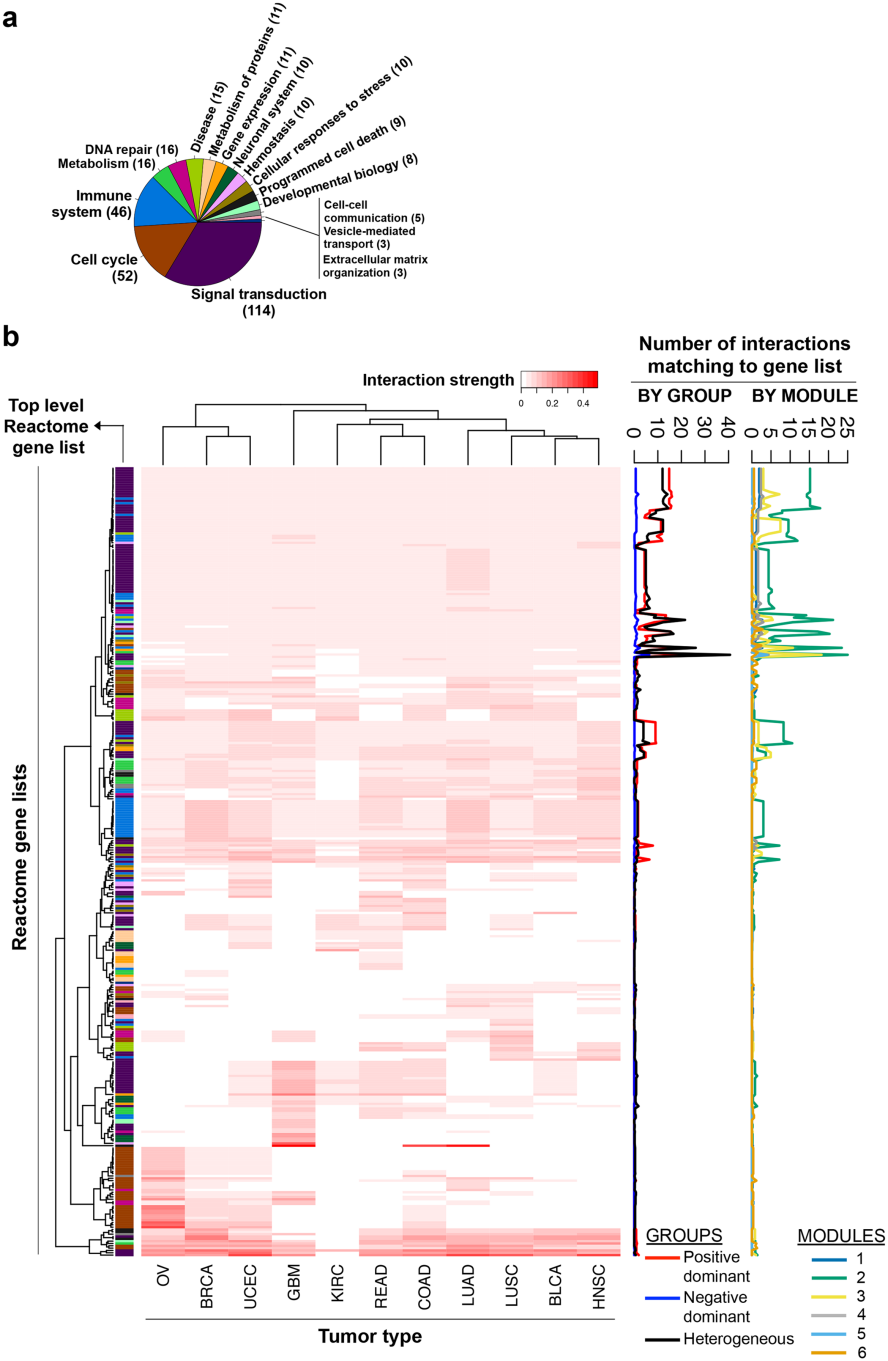
Mapping discovery set interactions to Reactome gene lists. 339 Reactome gene lists had a match with discovery set interactions. **A)** The number of gene lists among 339 that can be traced back to each top-level event. **B)** The average interaction strengths per tumor type for the 339 gene lists. The top-level Reactome event for each gene list is annotated on the left of the heatmap with colors adopted from A. Heat map orders for gene lists (rows) and tumor types (columns) are both obtained from Ward-linkage Euclidean-distance hierarchical clustering. The number of significant (consensus rank < 425) interactions that match to each gene list is broken down by module or group, averaged over tumor types, and tracked on the right of the heatmap. The numbers for the ‘module’ lines may be zero if the matching interactions are inter-module (linking two modules).

The universe of Reactome gene lists is not a flat structure, but a hierarchy. The top level of the hierarchy for human-specific gene lists consists of 24 biological processes according to Reactome Pathway Browser (S7 Fig). We first performed a parent-child analysis for the 339 gene lists in S4 Table. 16 out of 339 were one of the top-level (most general) biological processes, whereas 323 gene lists were child gene lists at different depths of the hierarchy (S5 Table). An analysis of the parents of the 339 gene lists revealed that the top-level ‘events’ **signal transduction**, **cell cycle**, and **immune system** had the greatest number of child gene lists (114, 52, and 46 respectively) that matched to at least one interaction in a tested tumor type (Fig 8A). This analysis also showed that more gene lists with positive ‘average interaction strengths’ were shared across tumor types than those specific for one or two tumor types (Fig 8B). In the Fig 8B heat map, we also tracked the number of significant (consensus rank < 425) interactions that match to each gene list broken down by module or group, and averaged over tumor types (S11 Table). Module 2 and 3 have the highest numbers of interactions that match the shown 339 Reactome gene lists, and these interactions are predominantly from the heterogeneous and the positive dominant groups (Fig 8B).

**Fig 9:**
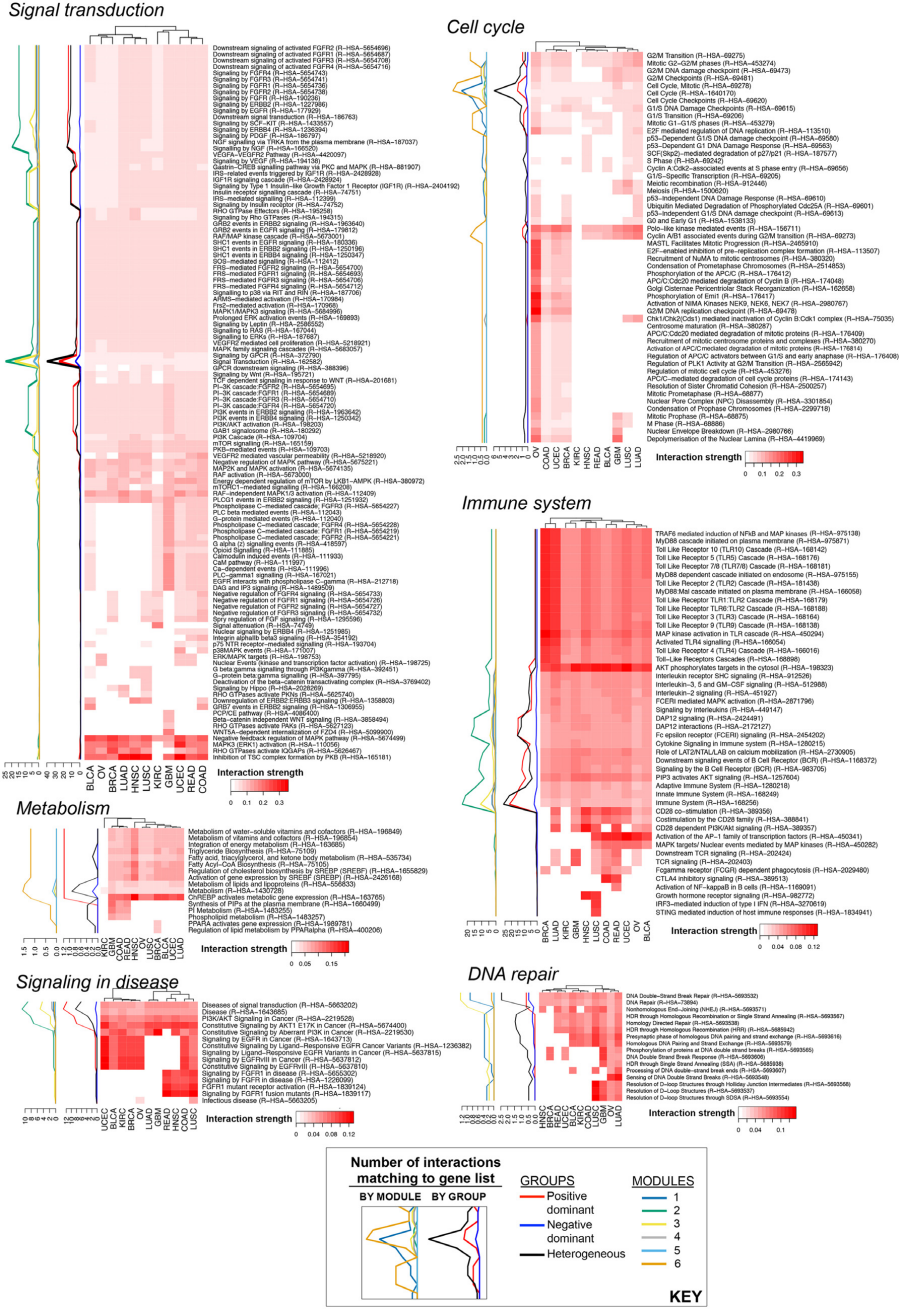
Pan-cancer similarities and differences for Reactome top-level events with the highest numbers of matching gene lists. Per tumor type average interaction strength for the top-level events 1) signal transduction, 2) cell cycle, 3) immune system, 4) metabolism, 5) DNA repair, and 6) disease are shown. The number of significant (consensus rank < 425) interactions that match to each gene list is broken down by module or group, averaged over tumor types, and tracked on the left of the heatmap. Heat map orders for gene lists (rows) and tumor types (columns) are both obtained from Ward-linkage Euclidean-distance hierarchical clustering. The numbers for the ‘module’ lines may be zero if the matching interactions are inter-module (linking two modules).

The 11 tumor types show strong similarities in terms of **signal transduction** interaction strengths (Fig 9 left panel). The signal transduction gene lists common to all tested tumor types include signaling by RTKs such as fibroblast growth factor (FGF) receptor family, epidermal growth factor (ErbB) receptor family, platelet-derived growth factor (PDGF) receptor family, vascular endothelial growth factor (VEGF) receptor family, insulin receptor family as well as signaling by G-protein-coupled receptors (GPCR) and Wnt, AKT/mTOR, and RAS/MAPK pathways. The gene lists not common across tumor types include the Hippo signaling (specific to LUAD, LUSC, and BRCA) and phospholipase C-related pathways (specific to COAD, READ, UCEC, GBM, KIRC, BLCA). However, the gene lists not common across tumor types are associated with very few matching interactions as indicated by the ‘group’ membership track on the left of the heatmap. It is possible that one interaction matches with multiple similar Reactome gene lists and populates the heatmap. The **disease** top-level event recapitulates the above signal transduction-related findings as about twenty interactions from Module 2 positive dominant group, and Module 3 heterogeneous group match with these gene lists (Fig 9 middle bottom panel, labeled as ‘signaling in disease’ as the majority of the gene lists are associated with signaling).

All tested tumor types exhibit moderate interaction strengths for cell cycle related gene lists (Fig 9 middle top panel). Most of these interactions are heterogeneous group edges from Module 5 and 6 as indicated by the group and module tracks. Carcinomas of the ovary, uterus, and breast, and adenocarcinoma of the colon have higher interaction strengths compared to other tumor types for several anaphase and metaphase-related gene lists such as those involving anaphase promoting complex (APC/C), its inhibitor Emi1, NIMA family kinases, and nuclear mitotic apparatus (NuMA). Other biological processes common to all tested tumor types are innate and adaptive immune signaling, metabolism, and DNA repair, as expected with transformed cells and the immune cells infiltrating the tumor microenvironment (Fig 9 middle and right panels). Interestingly, interactions that match to ‘metabolism’ gene lists are predominantly positive dominant group edges from Module 6. The ‘immune system’ gene lists match mostly with Module 2 edges that are from the heterogeneous or positive dominant groups. ‘DNA repair’ gene lists match with both Module 2 and 3 edges, albeit predominantly from the heterogeneous group. Other Reactome top-level ‘events’ that match to discovery set interactions include apoptosis, extracellular matrix organization, and cell-cell communication (S8 Fig).

## Discussion

Discovering PPIs in cancerous cells is an important but challenging goal. In this study, we computationally inferred proteomic networks in 11 human cancers using 13 different methods, and presented a performance comparison of the methods accepting a simplified reference network from the Pathway Commons information resource, which is based on experiments and publication digests, as the standard of truth. Pathway Commons is a collection of curated interactions from many different normal and disease conditions (a formal and computable representation of available pathways and interactions). We acknowledge that a complete standard of truth for pathways is not currently available and that our methodology is therefore subject to certain caveats, as discussed below. Despite these caveats, computational inference of PPI networks from measurements of protein levels across a set of conditions are attractive in that they can reduce the hypothesis space of interactions and inform researchers on the potentially active pathways in the experimental model.

Our comparison of the performance of network inference methods indicates that no single method has the best performance in all tumor types, but a group of six methods, including diverse techniques such as correlation, mutual information, and regression, consistently rank highly among the tested methods. These six methods consist of *RIDGENET* and *LASSONET*, ridge and lasso regression-based partial correlation methods employing an ℓ_2_ and ℓ_1_ penalty respectively; *ARACNE-A*, *ARACNE-M*, and *CLR*, mutual information methods that differ based on their penalty type or the choice of standardization for mutual information; and SPEARMAN CORRELATION, which assesses the strength of the linear relationship between the *ranks* of the values in two same-length vectors. From a tumor-type perspective, we find that not all tumor types are equally amenable to network discovery with RPPA data. Five tumor types (KIRC, OV, COAD, READ, and BLCA) consistently had lower AUPR predictions by all network inference methods.

In a recent multi-method comparison study for gene network inference, regression-based methods were represented mostly by modifications of the ℓ_1_-penalized lasso algorithm; however methods involving an ℓ_2_ penalty, such as ridge regression or elastic net, were not included [35]. Moreover, the ℓ_1_-penalized methods did not achieve the best overall performance in gene network inference. We find in this study that ℓ_2_-penalized methods such as ridge regression can outperform the lasso in the inference of proteomic networks. Even though the concurrent execution of feature selection and model fitting may appear to be an attractive property for lasso regression, we recommend performing an unbiased test for both ℓ_1_ and ℓ_2_-penalized models in the exploratory phase of a study. It is not guaranteed that the variables selected by the ℓ_1_ penalty will be the most biologically important ones in the system.

A network of 1008 most significant interactions inferred by high-performing methods reveals that these interactions can be grouped into three. The group termed ‘positive dominant’ contains mostly positive interactions with generally high weights. Nine interactions that exist in at least 10 of the 11 tumor types with very strong consensus weights are also in this group (S1A Table), and potentially occur due to cross-reactivity of the antibodies. The other two groups are termed the ‘negative dominant’ (generally negative weights), and the ‘heterogeneous’ groups. The network of 1008 edges contains four robust densely connected modules, two of which do not include any edges from the negative dominant group (Modules 2 and 4). Strikingly, the ratio of phosphospecific antibodies in one of these two modules is 91% (Module 2). While it is possible that a technical bias may be leading to high correlations between antibodies of the same type, a biological reason such as the coordinated regulation of signal transduction events may also be strongly contributing to the Module 2 interactions between phosphospecific antibodies because phosphospecific and non-phosphosphospecific antibodies may exhibit a high number of interactions as in Module 1. The positive dominant and heteogeneous groups are scattered, albeit unevenly, to the four robust modules. Mapping these 1008 most significant interactions to Reactome gene lists confirms the pan-cancer importance of signal transduction pathways, innate and adaptive immune signaling, cell cycle, metabolism, and DNA repair; and also suggests several gene lists that may be specific to a subset of tumor types.

We observe a paucity of negative dominant group edges that match with Reactome gene lists (Figs 7 and 8). A possible reason may be that negative correlations in our study imply mutual exclusivity, not inhibitory relationships. Reactome gene lists are not designed to group together mutually exclusive proteins unless there is a flow of influence (*i*.*e*. activation/inhibition) from one to the other. It is also not implausible that an **inhibitory** event, such as phosphorylation of protein A by protein B, leads to a positive correlation between A and B as their concentrations may increase or decrease together.

The caveats in our workflow, as shown in Fig 1, concern both the *computational inference* and the *pathway knowledgebase* arms of the analysis. In the computational inference arm (Steps 1 and 2), the caveats include questions regarding (1) the quality of RPPA experiments and whether the signal-to-noise ratio in RPPA experiments is high enough to allow the inference of direct interactions, and (2) the reliability of results from computational network inference methods (S1A Text). In the pathway knowledgebase arm (Steps 3–5), the fidelity of pathway models in knowledgebases is limited due to factors including (1) the quality of wet-lab experiments for PPIs such as yeast-2-hybrid [34], (2) missing or inaccurate information in the database due to poor curation, (3) the lack of context information for PPIs, such as cell or tissue type or physiological conditions, and (4) the ascertainment bias in the knowledgebase (primarily incomplete coverage) as discussed in the Introduction. More generally, pathways in knowledgebases such as Pathway Commons are only model descriptions of reality typically summarizing a set of experiments and do not represent an absolutely ‘true’ (and certainly not complete) set of interactions.

In future work, it will be important to assess the predictive power of the inferred PPI networks. For example, it would be useful to evaluate these networks in terms of how much they assist in the understanding of oncogenesis, response to therapy, and design of combination therapies that deal with feedback loops. It is also desirable to incorporate time-dependent readouts from perturbation experiments to be able to build causal models and enhance the predictive power of proteomic networks. An obstacle against building causal models, such as Bayesian networks, with the PANCAN11 RPPA data was the relatively large size of the network (187 nodes, i.e. antibodies) compared with the number of available samples in individual tumor types (between 127 and 747). Probabilistic models such as Bayesian networks require the number of samples to be at least an order of magnitude larger than the number of nodes for a sound estimation of model parameters [36].

The significance of this work extends beyond cancer. Discovering direct, potentially causal interactions between proteins is an opportunity in all areas of molecular biology where proteins are measured in different conditions and where correlations are informative. The methodology presented here can easily be adopted to study interactions in different molecular biology contexts.

## Methods

### Dataset

The pan-cancer reverse phase protein array (RPPA) dataset was downloaded from ***The Cancer Proteome Atlas***[5] on April 12, 2013. This dataset is denoted as PanCan11 and contains protein expression data for 3467 tumor samples and 187 antibodies, 51 of which are phosphospecific and 136 of which are non-phosphospecific. The 11 tumor types represented in this dataset are bladder urothelial carcinoma (BLCA), breast invasive carcinoma (BRCA), colon adenocarcinoma (COAD), glioblastoma multiforme (GBM), head and neck squamous cell carcinoma (HNSC), kidney renal clear cell carcinoma (KIRC), lung adenocarcinoma (LUAD), lung squamous cell carcinoma (LUSC), ovarian serous cystadenocarcinoma (OVCA), rectum adenocarcinoma (READ), and uterine corpus endometrioid carcinoma (UCEC).

PanCan11 patient samples were profiled with RPPA in different batches, and normalized with replicate-based normalization (RBN). RBN uses replicate samples that are common between batches to adjust antibody means and standard deviations so that the means and standard deviations of the replicates become the same across batches.

### Pathway Commons query with PERA

Pathway Commons stores pathway information in BioPAX[22] models that contain formal computable representations of diverse events such as biochemical reactions, complex assembly, transport, catalysis, and physical interactions. We queried Pathway Commons with the "prior extraction and reduction algorithm" (PERA)[26] for the proteins and phosphoproteins in the PANCAN11 RPPA dataset.

PERA is a software tool and a protocol that, given a set of observable (phospho- and/or whole) proteins, extracts the direct and indirect relationships between these observables from BioPAX-formatted pathway models[26]. PERA accepts a list of (phospho)proteins identified by their HGNC symbols, phosphorylation sites and their molecular status (one of ‘active’, ‘inactive’, or ‘concentration’) as input and, based on the pathway information provided by the Pathway Commons information resource[20], it produces a binary and directed network. The major advantage of PERA over other similar tools, such as STRING[37] or GeneMania[38], is that it considers not only the name/symbol of a protein but also its phosphorylation sites, enabling finer mapping of entities and pathways.

We downloaded the web ontology (OWL) file for Pathway Commons version 2 on 10/1/2013, and implemented PERA v2.9.1 with the following command:

java -Xmx3g -jar bpp291.jar \

 -l 1 –t 3 \

 -o output.tsv input.tsv \

 pathwaycommons2.owl

The PERA input file (*i*.*e*. input.tsv) is provided as S6 Table. The–l value of 1 determines that PERA will output an interaction between two entities only if the distance between them is 1, i.e. there is no intermediary entities. The–t value of 3 determines that the phosphorylation site mismatch tolerance is 3. For instance, if a PERA input includes phosphorylation site S473 for Akt, PERA will consider all interactions in the residue range (470, 476) for this phosphoprotein. The post-processing of the PERA output file included two steps: 1) As the network inference methods employed in this study produce only undirected network predictions, we first converted the directed network in the PERA output to an undirected network. 2) We then removed any existing duplicate and/or self edges before using this network as a gold standard in performance evaluation.

### Implementing network inference and community detection methods in R

The analysis was performed using the R language[39]. The R functions used to implement the network inference methods are as follows: The cor function in the **stats**[40] package for PEARSONCOR and SPEARMANCOR; the ggm.estimate.pcor and cor2pcor functions in the **GeneNet**[41] package for GENENET and SIMPLEPARCOR; the ridge.net, pls.net, and adalasso.net functions in the **parcor**[42] package for RIDGENET, PLSNET, and LASSONET; the glasso function in the **glasso**[43] package for GLASSO; the aracne.a, aracne.m, clr, and mrnet functions in the **parmigene** package[44] for ARACNE-A, ARACNE-M, CLR and MRNET. The ELASTICNET method was implemented as a modification of the adalasso.net function in the **parcor** package, and is available upon request. Mathematical descriptions of the algorithms used are provided in S1F Text.

Community detection algorithms were implemented with the cluster_fast_greedy, cluster_spinglass, cluster_infomap, cluster_louvain, and cluster_walktrap functions in the **igraph**[45] package version 1.0.1.

### Evaluating performance of network inference methods

The steps involved in computational network prediction and performance evaluation are discussed in detail in S1E Text. Here we discuss the construction of precision-recall curves. *Precision* was defined as the fraction of the number of correctly predicted edges (predicted edges that can be found in the gold standard) to the number of all predicted edges. *Recall* was defined as the fraction of correctly predicted edges to the number of all edges in the gold standard.

The PR curve for a given parameter configuration was constructed by taking the edge list ranked from the most significant to the least, and then iterating over the edges so that we obtained, at each iteration, a cumulative edge set that included all the edges seen up to and including that iteration. For each iteration, we computed the precision-recall value pair for the edge set and placed this value pair on the PR plot. We plotted a separate PR curve for each parameter configuration for the nine methods that required specification of parameter values (all methods except *PEARSONCOR*, *SPEARMANCOR*, *SIMPLEPARCOR*, and *GENENET*). The PR curve that had the greatest area under the curve (AUPR) between the [0,0.1] recall range (i.e. limited-recall) was identified as the optimal PR curve for that particular method. The optimal parameter values for the limited-recall case are shown in S5 Fig. For methods that did not have user-specified parameters, there was only one PR curve and that was adopted as the optimal PR curve. In the subsequent step, the AUPRs from the optimal PR curves were compared in order to rank the methods and evaluate their performance relative to the gold standard network.

### Rationale to prefer high precision over high recall

We find that the inferred interactions in various tumor types are a relatively small subset of the gold standard network derived from Pathway Commons (*i*.*e*. low recall). Low levels of recall are readily acceptable for satisfactory performance because it is expected that interactions inferred from a single disease (cancer) and a single cancer type will not retrieve all of the interactions in the gold standard. However, it is desirable that, when an algorithm calls an interaction, there is a high probability that this inference is correct, *i*.*e*. high levels of precision are essential for nominating a network inference method as competitive.

### Mapping discovery set edges to Reactome gene lists

Three data files were downloaded from the Reactome website http://www.reactome.org/pages/download-data/: 1) Reactome Pathways Gene Set (S7A Table) on 11/11/2015, 2) Complete list of gene lists (ReactomePathwayLabels.txt) on 11/16/2015, and 3) gene list hierarchy relationship (ReactomePathwaysRelation.txt) on 11/16/2015. The first file contained a total of 1705 gene lists. The second file contained gene list labels, unique Reactome identifiers, and species information. The third file contained the unique identifiers of parent gene lists adjacent to those of the child gene lists. The ID in the left column was one level above, in other words a superset of the ID in the right column. Reactome identifiers also include characters to denote the species information. The information in the second and third files was used to filter out non-human gene lists (S7B and S7C Table respectively). After the removal of duplicate entries, the number of human-specific gene lists in file 2 was 1869. The overlap between these 1869 human-specific gene lists and the 1705 gene lists in file 1 was 1669, which was used as a gold standard in the subsequent mapping analysis.

The PERA input in S6 Table lists the gene(s) that correspond to each antibody used in this study. The complete list of these genes including all paralogs is provided in S8 Table, and contains 167 uniqe genes. In mapping the discovery set interactions to Reactome gene lists, each interaction is represented by the gene(s) corresponding to the interaction partners. The pseudocode for this mapping is as follows:

# Constants

N = 11 # Number of tumor types

T = 425 # The threshold for consensus edge rank

R = 1669 # The number of gene lists in the Reactome

 # human gold standard

Initialize P # 1669 by 11 matrix that stores the

 # ‘average interaction strength’ of each

 # Reactome gene list for a given tumor type

for i = 1:N # Tumor types

 q <- Number of edges in tumor type i that pass consensus rank threshold T

 Initialize M # 1669 by q matrix that maps the most

  # significant interactions in tumor type

  # i to Reactome gene lists

 for j = 1:q # Interactions

  1. Identify the corresponding gene(s) for each interaction partner. Let these be set1 and set2.

  2. If set1 and set2 has a non-empty intersection, this is a self interaction. Skip to next interaction.

  3. Otherwise

   for k = 1:R # Reactome gene lists

    4. If set1 and set2 both have at least one member in gene list k, this is a match. Assign absolute weight to entry M[k,j]. This weight is the consensus edge weight penalized (divided) by the number of genes in gene list k.

   end

  end

  5. Average the q interactions (only the real-valued ones) for each one of the 1669 gene lists. This is the **average interaction strength** for a given Reactome gene list in a given tumor type.

  6. Insert the vector created in **Step 5** as a column in matrix **P**

**end**

### Availability

Networks from the TCGA RPPA or tab-delimited user data can be inferred and visualized with the **ProtNet** web application at http://www.sanderlab.org/protnet (A tutorial is provided in S1 Protocol).

R scripts used in the analysis are provided in S2 Protocol.

## Supporting Information

S1 Text **A) Major caveats in performance evaluation of computational proteomic network predictions. B)** Different tumor types are not equally amenable to network discovery with RPPA data. **C)** The relative merit of TOP6 and ALL13 consensus calls. **D)** Choosing a cutoff for consensus edge ranks in tumor type clustering. **E)** Steps involved in computational network inference. **F)** Mathematical descriptions of the 13 tested methods.
(DOCX)

S1 Fig Statistics of inferred networks for each tumor type. **(A,C,E,G)** The x-axis shows tumor types while the y-axis shows **(A)** average node degree, **(C)** network density, **(E)** optimal number of modules, and **(G)** highest modularity score. Colors denote different methods as shown in the legend. **(B,D,F,H)** Scatter plots of these network statistics with AUPR.
(PDF)

S2 Fig Statistics of inferred networks for each method. In contrast with S1 Fig, methods are shown on the x-axis and colors denote different tumor types. The y-axis shows **(A)** average node degree, **(B)** network density, **(C)** optimal number of modules, and **(D)** highest modularity score.
(PDF)

S3 Fig The comparison of TOP6 and ALL13 edge lists. First, a pan-cancer rank for each edge is computed separately from the TOP6 and the ALL13 methods. Edge lists ordered according to these pan-cancer ranks are then compared. **(A)** Overlap and difference percentage between the significant ALL13 and TOP6 edges as the significance threshold is varied from 1 to 3000. **(B)** Precision of the significant ALL13 and TOP6 edges in the gold-standard network as the significance threshold is varied from 1 to 3000.
(PDF)

S4 Fig Determining the edge rank cutoff for tumor-type clustering. **(A)** The left y-axis shows the mean of the percentages of shared edges. The **red** dotted line is for the mean from all pairwise tumor-tumor comparisons. The **black** dotted line is for the mean from the 11 tumor-random comparisons each of which involve one distinct tumor type and the same randomly ordered edge list. The right y-axis shows the number of edges in the union set from all tumors (**blue** line). The x-axis shows the cutoff value for the TOP6 consensus edge ranks. **(B)** The left y-axis shows the percentage of variance explained by PC1, PC2 and PC3 (**black, red,** and **orange** dotted lines respectively). The right y-axis indicates the sum of the variance percentages explained by the first three principal components (**blue** line). The x-axis shows the cutoff value for the TOP6 consensus edge ranks.
(PDF)

S5 Fig Optimal parameter values in the limited-recall case for each method and tumor type. (PDF)

S6 Fig The number of edges at limited recall for each method and tumor type. The high-performing TOP6 group is shown in the top panel, and the other methods in the bottom panel. All 13 methods are used for the PCA of methods, but only the TOP6 methods are used for the PCA of tumor types.
(PDF)

S7 Fig Reactome event hierarchy. Top-level Reactome gene lists are annotated on the network with connections to child gene lists. Figure adopted from Reactome Pathway Browser.
(PDF)

S8 Fig Pan-cancer similarities and differences for Reactome top-level events not shown in Fig 9. Per tumor type average interaction strengths for ten Reactome top-level events are shown. The number of gene lists for each top-level event is provided in Fig 8A. The number of significant (consensus rank < 425) interactions that match to each gene list is broken down by module or group, averaged over tumor types, and tracked on the left of the heatmap. Heat map orders for gene lists (rows) and tumor types (columns) are both obtained from Ward-linkage Euclidean-distance hierarchical clustering. The numbers for the ‘module’ lines may be zero if the matching interactions are inter-module (linking two modules).
(PDF)

S1 Table The list of 1008 discovery set interactions. **A)** For each interaction, consensus weights in the 11 tested tumor types, pan-cancer recurrence, pan-cancer weight, group and module information, and the matching Reactome gene lists are provided. **B)** For each interaction, binary values are provided to indicate whether the consensus edge rank in the relevant tumor type was smaller (more significant) than 425. The sum of each row corresponds to the pan-cancer recurrence field in **A**.
(XLSX)

S2 Table Consensus for community detection algorithms. **A)** 186 x 186 matrix of frequency values for the Fig 6 heatmap. **B)** The assigned modules for 186 antibodies.
(XLSX)

S3 Table The interactions of phosphospecific antibodies in Module 1. Only 14 out of 64 interactions are between two phosphospecific antibodies.
(XLSX)

S4 Table Per tumor-type average interaction strengths for the 339 Reactome gene lists that match with at least one interaction in the discovery set. (XLSX)

S5 Table Reactome gene list IDs for the 339 gene lists in S4 Table, and the corresponding parent labels until the top level of the hierarchy. (XLSX)

S6 Table PERA input file. PERA queries Pathway Commons using the gene symbol, type, and phosphorylation site fields. The antibody name field is a placeholder for labels and does not require standard names. The antibody number field has to be removed when running PERA.
(XLSX)

S7 Table Downloaded Reactome data files. **A)** 1705 Reactome gene lists before filtering out non-human gene lists. **B)** Reactome IDs and labels for human gene lists. **C)** The parent-child information for Reactome IDs of human gene lists. The ID in the left column is one level above, in other words a superset of the ID in the right column.
(XLSX)

S8 Table 167 unique genes. 187 antibodies in this study target proteins that correspond to a total of 167 unique genes taking into account the multiple isoforms of proteins (*i*.*e*. multiple paralogs for the genes).
(XLSX)

S9 Table The M matrix used in principal components analysis of network inference methods. This matrix was constructed as explained in Step 5 of S1E Text.
(XLSX)

S10 Table Consensus ranks, weights, and signs for limited recall edges. The methodology for obtaining the values is described in Step 6 of S1E Text. **A)** Bladder carcinoma (BLCA), **B)** Breast carcinoma (BRCA), **C)** Colon adenocarcinoma (COAD), **D)** Glioblastoma multiforme (GBM), **E)** Head and neck squamous cell carcinoma (HNSC), **F)** Clear cell renal cell carcinoma (KIRC), **G)** Lung adenocarcinoma (LUAD), **H)** Lung squamous cell carcinoma (LUSC), **I)** Ovarian carcinoma (OV), **J)** Rectal adenocarcinoma (READ), **K)** Uterine corpus endometrial carcinoma (UCEC).
(XLSX)

S11 Table Average number of matching interactions for each Reactome gene list. For each tumor type, significant interactions are determined based on consensus rank threshold of 425. The number of interactions that match to Reactome gene lists is counted for each module and group. Module and group counts are averaged across 11 tumor types and plotted in Figs 8 and 9 tracks next to heatmaps.
(XLSX)

S1 Protocol Tutorial for the ProtNet network inference and visualization web tool. (PDF)

S2 Protocol A compilation of R code used to obtain the results in this study. (ZIP)
