# Supplementary material for: A Multi-Method Approach for Proteomic Network Inference in 11 Human Cancers
Source: PLoS Comput Biol. 2016 Feb 29;12(2):e1004765. doi: 10.1371/journal.pcbi.1004765 (PMC4771175; doi:10.1371/journal.pcbi.1004765)
Supplement: S1 Text — A) Major caveats in performance evaluation of computational proteomic network predictions. B) Different tumor types are not equally amenable to network discovery with RPPA data. C) The relative merit of TOP6 and ALL13 consensus calls. D) Choosing a cutoff for consensus edge ranks in tumor type clustering. E) Steps involved in computational network inference. F) Mathematical descriptions of the 13 tested methods. (DOCX) [file pcbi.1004765.s001.docx]

**SUPPLEMENTARY INFORMATION**

**S1A Text: Major caveats in performance evaluation of computational proteomic network predictions**

**The pros and cons of using RPPA data in the inference of cellular networks**

The RPPA technology has previously been validated to allow a practical screening of potential biomarkers in certain cancers [[1](#_ENREF_1),[2](#_ENREF_2)]. However, using RPPA datasets for cellular network inference has both **pros** and **cons** when compared with using mRNA-measuring platforms such as RNA-seq or microarrays. The pros are primarily related to cellular physiology and include facts such as following: (1) Proteins are closer to the organism’s **phenotype** than mRNAs; (2) mRNAs are subject to **degradation**, so correlation between mRNA and protein abundance is low; (3) mRNAs cannot be used to measure activity levels of phosphorylated proteins which are key components of signal transduction, an important mechanism for the flow of influence/ information in the cell. Collectively, these factors suggest that RPPA levels are a better proxy for both protein abundance and pathway activity levels compared with mRNA data.

The **cons** of using RPPA experiments for network inference primarily involve technology and platform related shortcomings. For instance, (1) the RPPA platform may have higher technical noise than transcriptomic profiling platforms such as RNA-seq and mRNA microarrays. (2) Currently, there are antibodies for only a limited number of proteins (less than 300); but it is possible to perform genome-wide mRNA expression profiling using RNA-seq or microarrays. (3) Some of the widely used antibodies are known to lack specificity for their conjugate proteins (for instance, antibodies for phospho-EGFR and phospho-HER2 are likely to bind to both proteins); and some others may have yet under-appreciated specificity problems. (4) The quality of antibodies has to be checked right before an RPPA experiment because the specificity of antibodies can decay over time. Also, (5) quantifying the abundance of the non-phosphorylated state of a protein currently remains a challenge in RPPA experiments due to the lack of an antibody that specifically targets the non-phosphorylated state.

**The community-wide underperformance of network inference methods**

High-throughput genomic and proteomic platforms generate high-dimensional datasets with a strong correlation structure. The accurate classification of these correlations into direct and transitive interactions is an unsolved challenge.

The precision of computational network inference methods remains less than satisfactory not only for PPI networks but also for other applications such as gene regulatory networks [[3](#_ENREF_3)]. One important reason could be that, the statistical dependence paradigms involving measures such as partial correlation and mutual information are not sufficient to model the biochemical reactions in the cell. Moreover, the multi-protein interactions in many cellular processes constitute a level of complexity higher than can be modeled by pairwise interactions alone. However, the ansatz is that predominant pathway activities can potentially be captured by the inference of direct pairwise interactions.

Further, to what extent one can empirically characterize direct and transitive relationships depends substantially on the inclusion of all potentially confounding variables in the model. A regulatory mechanism such as proteolysis, epigenetic silencing, microRNA regulation, etc. that concurrently acts on two proteins, but is not accounted for in the dataset could easily lead to **spurious direct-association calls** between the proteins. On the contrary, it is possible that true direct assocations could be missed due to tight regulation among a group of proteins: for instance, high correlations between a transcription factor and multiple proteins it upregulates could hinder the inference of one or more of the direct interactions between the transcription factor and each one of the regulated proteins.

Such lack of signal resolution in tracking information/influence flow in biological networks can be compensated by using information from time-series and perturbation experiments, i.e. temporal and causal domain respectively. However, it is important to note that strong correlations found in the temporal domain alone, or found in the causal domain alone are not necessarily reproducible in the other domain. For instance, it was shown that, in experiments with receptor tyrosine kinases, some phosphosite pairs were strongly correlated in the temporal domain (i.e., having similarly shaped time courses) but not the causal domain (i.e. not responding similarly to shRNA perturbations); whereas other phosphosite pairs were not well correlated in the temporal domain (i.e. their time course shapes look different), but were correlated in the causal domain (i.e. they respond similarly to perturbations) (J. Wagner, personal communication, 2014). These two phenomena are exemplified by the [phospho-PKC-beta & phospho-MARCKS], and the [phospho-PKC-beta & phospho-ERK] pairs respectively. These results suggest that even time-course data, let alone static profiles, may not be sufficient to infer causal relationships among proteins in the cell. However, static computational PPI network predictions are still attractive in that they can reduce the hypothesis space of interactions and prioritize them so that interactions with higher statistical significance can functionally be studied in the laboratory before others.

**S1B Text: Different tumor types are not equally amenable to network discovery with RPPA data.**

We see in **Fig 3A** that none of the methods reach an AUPR level of 0.02 for five of the eleven tumor types (KIRC, OV, COAD, READ, and BLCA). For three other tumor types, namely LUAD, HNSC, and LUSC; mutual information and correlation family methods *ARACNE*, *CLR, SPEARMANCOR,* and *PEARSONCOR* achieve some of the highest AUPRs in this study. On the other hand, regularized methods such as *RIDGENET, GLASSO,* and *ELASTICNET* have some of the worst performances for these tumor types. This is a surprising result because *SPEARMANCOR* and *PEARSONCOR* do not remove any transitive edges from the system, and *RIDGENET* is shown to be the best-overall performing method in **Fig 3B**. There is an inherent difference in LUAD, LUSC, and HNSC datasets that enables non-regularized methods to perform better. We asked whether a difference between regularized and non-regularized methods might be leading to differences in the topological characteristics of predicted networks, that in turn could partially explain the higher performance of non-regularized methods in the LUAD, LUSC, and HNSC datasets. One such difference is the number of conditioned nodes when the independence of two nodes is examined. Regularization-based methods condition on the other N-2 nodes when inspecting the existence of an edge in a system with N nodes. In contrast, non-regularized methods either do not condition on any nodes at all (i.e. *correlation*) or condition on only a third node (i.e. *ARACNE*).

We investigated the topological characteristics of the predicted networks and the gold-standard benchmark by computing network statistics such as average-node-degree, network density, optimal number of modules, and modularity index. The *average-node-degree* for an undirected network is the mean value of all node degrees where the node degree is simply the number of edges incident on a node. The *density* of a network is the ratio of the number of edges in the network to the maximum possible number of edges. The *optimal number of modules* and the *modularity indices* are computed using the edge-betweenness statistic in the Girvan-Newman algorithm [[4](#_ENREF_4)].

We observe in **S1B Fig** and **S1D Fig** that the average-node-degree and network-density in predicted networks have a high negative correlation with AUPR (Spearman r = –0.626 and –0.453 respectively). Indeed, the methods with the highest values for these statistics in **S1A Fig** and **S1C Fig**, namely *SIMPLEPARCOR* and *PLSNET*, are among the poor-overall performing methods according to **Fig 3B**. In contrast, the best-overall performing methods such as *RIDGENET* and *ARACNE* are not the ones with the lowest density or the lowest average-node-degree network predictions. The high-performers tend to yield networks with density and average-node-degree values that have not substantially deviated from the same statistics derived from the gold-standard network (**S1A–S1D Fig**).

We note that regularized and non-regularized methods do not have a marked difference in terms of network density and average-node-degree. However, we observe in **S2C Fig** and **S2D Fig** that (1) low optimal module number and (2) high modularity score are both discriminating and also pan-cancer-wide characteristics of non-regularized methods such as *SPEARMANCOR*, *PEARSONCOR*, *ARACNE* and *CLR*. It can be speculated that these methods yield more modular networks because the number of conditioned nodes is far fewer (or zero), thus they preserve the original protein-protein correlation structure in the data to a greater extent. Preserving the original correlation structure is not always a desirable property since the goal in network construction is to eliminate transitive edges, *i.e.* spurious correlations. However, the correlation structure in the LUSC, LUAD, and HNSC datasets could be so strong that (1) it leads to better performance for non-regularized methods, and (2) it causes regularized methods to erroneously eliminate true edges due to conditioning on N-2 nodes when inspecting the existence of an edge. Considering the shared tissue or cell type of origin among LUSC, LUAD, and HNSC, the inherent difference in these datasets compared with the others in PANCAN11 potentially stem from a biological difference rather than a systematic bias.

**S1C Text: The relative merit of TOP6 and ALL13 consensus calls**

Each edge has a separate consensus rank from the TOP6 and the ALL13 groups of methods, computed as the average of the ranks from the relevant methods. Since this consensus rank is different in each tumor type, a pan-cancer consensus can be obtained by taking the average of consensus ranks from all 11 tumor types. It is possible to perform a pan-cancer comparison of TOP6 and ALL13 using these single pan-cancer edge ranks. We took the same number of top edges from the pan-cancer-ranked edge lists of TOP6 and ALL13, and computed (a) the overlap and difference percentages between the two lists, and (b) the precision score of each list for predicting the interactions in the gold-standard network. We then varied the number of edges from 1 to 3000 in order to assess the trends in these variables.

**S3A Fig** shows that there is a high level of intersection between these two sets: higher than 80% for the most restrictive edge-rank thresholds, dropping to 60% around 250 edges, and eventually plateauing around 70%. The TOP6 and ALL13 lists may have modest differences, but we see in **S3B Fig** that TOP6 edges achieve a slightly superior AUPR value compared with ALL13 edges when compared against the benchmark. The superiority in performance is more pronounced for the first 1000 edges after which the performances of the two groups become virtually equivalent.

**S1D Text: Choosing a cutoff for consensus edge ranks in tumor type clustering**

We would like to perform an unsupervised clustering of tumor types using only high-confidence edges that well represent both the similarities and the differences between tumor types. The union set of edges from limited-recall (**S6 Fig**) has 13193 edges, resulting in a network density of 76% (in the total of 17391 possible edges). Considering that the gold-standard network has a density of ~9% (**S1C Fig**), it is clear that a network with 76% density will have an extremely low true positive rate. Therefore, we sought to adopt a more stringent criterion in choosing the edge-rank cutoff for the edges to be used in tumor-type clustering.

Each edge in a tumor type has a consensus rank, computed as the average of ranks from the TOP6 methods. The consensus ranks of these edges are thresholded with the same cutoff value in all tumor types in order to determine the edges that will be used in the unsupervised clustering of tumor types. When the cutoff value is 25, the overlap of edge sets from different tumors is about 35% (computed as the average of the overlap percentages between each pair of tumors) (**S4A Fig**). This high overlap percentage indicates that the most significant edges in individual tumor types are largely shared across all tested tumor types. Indeed, **Fig 6** shows that the majority of these edges are interactions involved in signal transduction pathways that result in the growth and proliferation of neoplastic cells.

For a given rank-cutoff value, the union of edges from the 11 tumor types was obtained; and the consensus edge weights for these edges were used to cluster the tumor types. A principal component analysis along the tumor-type dimension was performed to infer the projections of the data with greatest separation power. This analysis revealed that the variance explained by the first 3 PCs started as high as 75% when the rank-cutoff value was low, but gradually declined as the cutoff was lowered (**S2B Fig**). In particular, the sum of the first three principal components made two local maxima at cutoff values 375 and 425 before showing a steadily declining trend. Therefore, we decided to perform unsupervised clustering of tumor types using the union of of edges with consensus ranks lower than 425 in the 11 tumor types. The number of edges in each tumor type that pass this criterion is given in **Table B in S1E Text** with the union set having 1008 edges (*i.e.* the “discovery set”). The interaction partners and consensus weights in this set are given in **S1A Table**.

**S1E Text. Steps involved in computational network inference**

**Step 1: Running the methods (by varying parameter values if applicable)**

The 13 network inference methods used in this study are shown in **Table A**. Nine of these methods require the user to specify at least one parameter value. The parameters and the tested values for the former nine methods are also listed in **Table A**. These parameter values were chosen to achieve a good representation of the parameter space, and when the parameter concerns sparsity, to cover the full spectrum from the most sparse networks to fully-connected ones.

Table A: The parameters and tested values for each network inference method. R expressions are given for the tested values if the number of values is greater than 5.

| **Family** | **Method** | **Parameter type** | **Tested values** |
| --- | --- | --- | --- |
| Correlation | PEARSONCOR | - | - |
|  | SPEARMANCOR | - | - |
| Partial correlation with inverse covariance | SIMPLEPARCOR | - | - |
|  | GENENET | shrinkage ($\lambda$) | $\lambda$ internally optimized |
|  | GLASSO | sparsity ($\lambda$) | seq(0,1,length=150) \ {0} |
| Partial correlation with regression | PLSNET | cross-validation (K), maximum number of components to consider (Ncomp) | K = {3,4,5,10,20}, Ncomp = 30 |
|  | RIDGENET | cross-validation (K), shrinkage ($\lambda$) | K = {3,4,5,10,20}, $\lambda$ internally optimized |
|  | LASSONET | sparsity ($\lambda$) | seq(0,1,length=150) \ {0} |
|  | ELASTICNET | cross-validation (K), weight for $\mathcal{l}_{1}$ penalty ($\alpha$), sparsity ($\lambda$) | K = {3,4,5,10,20}, $\alpha$ = seq(0.01,0.99, length=10), $\lambda$ internally optimized |
| Mutual information | ARACNE-A | nearest neighbor (K),  additive penalty ($\varepsilon$) | K = {2,3,4,5,6}, $\varepsilon$ = c(1e-5,1e-4, seq(0,0.2, length=100), seq(0.2,0.4,length=49)) \ {0,0.2} |
|  | ARACNE-M | nearest neighbor (K),  multiplicative penalty ($\tau$) | K = {2,3,4,5,6}, $\tau$ = c(1e-5,1e-4, seq(0,0.5, length=100), seq(0.5,1, length=49)) \ {0,0.5} |
|  | CLR | nearest neighbor (K) | K = {2,3,4,5,6} |
|  | MRNET | nearest neighbor (K) | K = {2,3,4,5,6} |

Of the remaining four methods, three do not have any user-specified parameters (*PEARSONCOR, SPEARMANCOR*, and *SIMPLEPARCOR*). The fourth one, *GENENET*, has a shrinkage parameter that is internally optimized in a data-dependent manner, hence not user-specified.

Technical details in running the algorithms include the following:

1. For *GLASSO* and *LASSONET*, $\lambda$ = 0 runs were removed because the algorithms did not converge. For *GLASSO*, we multiplied the output partial correlations by -1 as this operation was not performed by the R package.
2. For *PLSNET*, the maximum number of components for each regression model (Ncomp) was fixed at 30 because we empirically observed that the internally-optimized number of components for all (187 x 11) 2057 regression models (number of proteins * number of tumor types) was less than 30 in more than 99% of the cases.
3. For *RIDGENET*, the shrinkage parameter $\lambda$ was not varied in our tests because it is internally optimized and the value that minimizes the overall mean squared error across all cross-validation folds is used.
4. For *ELASTICNET*, we applied a grid search in each one of the regression models to find the [K,$\alpha$] pair that optimized the sparsity parameter $\lambda$. The number of regression models is the same as the number of proteins because a predictive model is built for each protein separately. Thus, there is not a [K,$\alpha$] pair optimal for all regression models in ELASTICNET; the optimal pair is different for each model. Since it is not possible to report all optimal [K,$\alpha$] pairs, we report in **S5 Fig** only the median across all regression models.
5. The optimal $\lambda$ in *ELASTICNET* was determined as the largest $\lambda$ in the $\lambda$ set whose cross-validation error was still within 1 standard error of the minimum cross-validation error (i.e. maximum predictive power).
6. For *ARACNE*, *CLR*, and *MRNET*, the mutual information between each pair of proteins was computed using entropy estimates from the K-nearest neighbor distances according to Kraskov *et al.* [[5](#_ENREF_5)]. Thus, we optimized K, the number of nearest neighbors, for the mutual information-based methods.

The output of the methods is a matrix of edge weights that we turn into a ranked edge list for subsequent analysis. With 187 proteins, the number of edges for a fully-connected network would be C(187,2) = 17391 edges. Sparse methods did not infer a weight for all 17391 edges, in which case the missing edges were assigned a weight of zero.

**Step 2: Plotting precision-recall curves for individual parameter configurations**

The precision-recall curve for a given parameter configuration was constructed by taking the ranked edge list from Step 1 and iterating over the edges from the most significant to the least significant. At each iteration, we obtained a cumulative edge set that included all the edges seen up to and including that iteration, and computed the precision-recall values for these edges. The precision-recall curve consisted of the results from all iterations. In the methods with user-specified parameters, a separate precision-recall curve was generated for each parameter configuration.

Sparse methods required an extra step in order to achieve a ranking of all 17391 edges. The ranks for the zero weight edges had to be artificially created, and were determined with a random permutation.

**Step 3: Computing AUPR and determining the optimal parameter values**

The area under a precision-recall curve (AUPR) was computed with the formula:

$$AUPR\left( \boldsymbol{p},\boldsymbol{r} \right)=\sum_{i=1}^{n-1} \frac{(p_{i+1}+p_{i})(r_{i+1}-r_{i})}{2}$$

where n indicates the number of edges in the edge list, p and r indicate precision and recall respectively, and the bold font **p** and **r** indicate the vector forms.

For the methods with user-specified parameters, the optimal parameter configuration was determined as the one that yielded the largest AUPR. In cases where a method had more than one parameter that needed optimization (*ARACNE-A, ARACNE-M* and *ELASTICNET*), we applied a grid search by computing the AUPR for each parameter combination, and then choosing the combination that had the largest AUPR.

As shown in **Fig 2A** and **Fig 2B**, we had two different sets of results based on (1) a **full-recall** case where we measured AUPR in the entire recall range from 0 to 1, and (2) a **limited-recall** case where we measured AUPR only in the [0,0.1] recall range. We report here (**S5 Fig**) the optimal values of the parameters only for the limited-recall case because of its superior performance as discussed in the main text and **Fig 2**. All subsequent analysis was performed with edges in the [0,0.1] recall range from the runs that used the optimal parameters in the limited-recall case.

**Step 4: Interpretation of edge weights and normalization for CLR**

The range of edge weights from methods in the correlation and partial correlation family is [-1,1]. The magnitude of the weight indicates the strength of the interaction while the sign shows the direction. In contrast, the mutual information family of methods do not infer the direction of edges, and output weights that are in the range [0,1]. The only exception to the [0,1] range is *CLR* (context-likelihood of relatedness), which has a lower bound of 0 but no upper bound as the test statistic (edge weight) in this method is essentially a z-score for mutual information values.

To ensure comparability between *CLR* edge weights and those of other methods, we normalized the *CLR* weights by their sum (separately in each tumor type) and multiplied by a coefficient to bring the range to [0,1] and also preserve the distribution of the original *CLR* weights. We observe in **Fig 3D** that *CLR* clusters together with other mutual information-based methods. This shows that the normalization procedure for *CLR* weights rendered *CLR* results comparable with other mutual information-based methods.

**Step 5: The principal component analysis (PCA) of the 13 network inference methods**

The optimal parameter configuration determined in Step 3 enabled us to determine the optimal edge weights from each method and for each tumor type. Using these optimal edge weights, we performed principal component analysis for the 13 network inference methods in the study.

To achieve this, we created a matrix ***M*** (**S9 Table**) where the methods were placed in the columns and the edge weights were placed in the rows. Edges from the tested tumor types were vertically stacked in order to preclude a tumor-type bias in the PCA results. The stack of each tumor type consisted of the union of limited-recall edges from all methods in that particular tumor type. For instance, the BLCA stack had 6959 edges as shown in the ***ALL13 union*** row in **S6 Fig**. The edges that were included in ***M*** but not found in the limited-recall set of an individual method were assigned a zero weight for that method, indicating that the limited-recall criterion imposed sparsity in the network. Constructed in this fashion, ***M*** consisted of 60202 rows (the sum of all columns in the ***ALL13 union*** row of **S6 Fig**) and 13 columns (**S9 Table**).

We then applied principal component analysis on ***M*** to discover its predominant features in the high-dimensional space formed by the linear combination of method vectors.

**Step 6: Computing the consensus ranks, weights, and signs for limited recall edges**

The weights assigned to edges and their ranks can vary considerably among the different methods. In order to determine the overall significance and weight of an edge across the methods (but within a given tumor type), we defined the *consensus edge ranks* and *consensus edge weights* respectively. The ***consensus rank*** of an edge is computed as the mean of its ranks from different methods. If one or more of the methods do not have that particular edge in their limited-recall sets, the rank for the edge is assigned to E + 1 where E is the *”total number of limited-recall edges inferred by the method”* (E does not include the random edges added to sparse methods in **Step 2**). This rank is not assigned to NA in order to ensure that all methods participate in the consensus rank calculation for that edge. However, the ***consensus weight*** of an edge is computed as the mean of its weights from different methods where the missing weights are assigned to NA.

Ranks were accepted as a proxy for significance. Thus, only the magnitudes of edge weights, and not the signs, were taken into account in computing the consensus ranks. Also, the sign for a consensus weight could not be derived from the average of the weights because mutual information-based methods always yield non-negative weights. Instead, the ***consensus sign*** for an edge was inferred from the sign of the Spearman correlation between the relevant antibodies.

The consensus ranks, weights, and signs for limited recall edges are provided separately for each one of the 11 tested tumor types in **S10A-K Table**.

**Step 7: PCA and hierarchical clustering on the 11 tumor types**

We used the consensus edge weights in the TOP6 methods to perform PCA and hierarchical clustering on the 11 tumor types. The design matrix, denoted with ***T***, had tumor types in the columns and edge weights in the rows. However, we restricted the edges to the ones that had TOP6 consensus-rank smaller (more significant) than 425. The preference of the TOP6 methods over ALL13, and the choice of the consensus edge-rank threshold of 425 were explained previously in **S1C Text** and **S1D Text** respectively. The number of edges that pass this threshold in each tumor type is shown in **Table B.** The union set of these highly-significant edges has 1008 elements and is referred to as the ***discovery set***.

Table B: The number of edges that have a consensus-edge-rank value less than 425 for each tumor type. Tumor types are listed in increasing order of the number of edges from left to right.

| **KIRC** | **LUAD** | **HNSC** | **BLCA** | **GBM** | **BRCA** | **UCEC** | **OV** | **LUSC** | **COAD** | **READ** | **Union** |
| --- | --- | --- | --- | --- | --- | --- | --- | --- | --- | --- | --- |
| 96 | 135 | 138 | 150 | 157 | 161 | 168 | 171 | 243 | 246 | 250 | 1008 |

We applied principal component analysis on ***T*** to discover its predominant features in the high-dimensional space formed by different tumor types. We also performed unsupervised clustering of the tumor types by applying hieararchical clustering on ***T*** with **Euclidean distance** and **Ward linkage**.

**S1F Text: Mathematical descriptions of the 13 tested methods**

**A) Correlation-based methods**

**A1) Pearson correlation**

The Pearson correlation between X and Y, denoted $r_{xy}$, is a measure of the strength of the linear relationship between X and Y; and can be given as:

$$r_{xy}=\frac{\sum_{i=1}^{n} \left( x_{i}-\overline{x} \right)\left( y_{i}-\overline{y} \right)}{(n-1)s_{x}s_{y}}$$

where $\overline{x}$ and $\overline{y}$ are sample means of X and Y, and $s_{x}$ and $s_{y}$ are the sample standard deviations of X and Y.

**A2) Spearman correlation**

The Spearman’s rank correlation coefficient is a non-parametric measure of statistical dependence between X and Y assessing the strength of the linear relationship between the **ranks** of the values in X and Y.

Let X^R^ and Y^R^ be the ascending ranks of the values in X and Y respectively, where ties are assigned a rank equal to the average of their positions. The Spearman correlation is computed from X^R^ and Y^R^ in the same way as the Pearson correlation:

$$r_{xy}=\frac{\sum_{i=1}^{n} \left( x_{i}-\overline{x} \right)\left( y_{i}-\overline{y} \right)}{(n-1)s_{x}s_{y}}$$

where, this time, $x_{i}$ and $y_{i}$ are individual components of X^R^ and Y^R^, $\overline{x}$ and $\overline{y}$ are sample means of X^R^ and Y^R^, and $s_{x}$ and $s_{y}$ are the sample standard deviations of X^R^ and Y^R^.

From here on, the data matrix is denoted with $X$. It contains n observations for p features, and the features are denoted with $X_{1}, X_{2}, \ldots, X_{p}$.

**B) Two formulations for partial correlation**

**Formulation 1: Inversion of the covariance matrix**

The p x p covariance (or correlation) matrix of the features can be inverted to obtain the p x p concentration (or precision) matrix denoted with $W$. Let $w_{ij}$ be the concentration value for row i and column j. Partial correlations $\rho_{ij}$ are then the negative of the standardized concentrations where the standardization is given as:

$$\rho_{ij}=\frac{w_{ij}}{\sqrt{w_{ii} w_{jj}}}$$

In graphical Gaussian models, the partial correlations represent the direct interactions between two variables, conditioned on all remaining variables.

**Formulation 2: Least Squares Regression**

Partial correlations can be obtained from any method based on a least squares regression. Following the exposé in Krämer *et al*. [[6](#_ENREF_6)], we consider the p linear regression models

$$X_{i}=\sum_{j\neq i} \beta_{j}^{(i)}X_{j}+\varepsilon$$

for $i=1,\ldots,p$ where $\varepsilon$ stands for i.i.d noise. The least squares estimate for the regression coefficients in the i^th^ model ${\hat{\boldsymbol{\beta}}}^{\boldsymbol{(i)}}=(\hat{\beta}_{1}^{(i)},\ldots,\hat{\beta}_{i-1}^{(i)},\hat{\beta}_{i+1}^{(i)},\ldots,\hat{\beta}_{p}^{(i)})$can be found with the optimization:

$${\hat{\boldsymbol{\beta}}}^{\boldsymbol{(i)}}=\arg\min_{\boldsymbol{\beta}\in\mathbb{R}^{p-1}} \left\| X^{(i)}-X^{(\backslash i)}\boldsymbol{\beta} \right\|^{2}$$

where $X^{(i)}\in\mathbb{R}^{n}$ is the i^th^ column of $X$, and $X^{(\backslash i)}\in\mathbb{R}^{n x (p-1)}$ is the matrix obtained from $X$ by deleting the i^th^ column. The partial correlation between protein $i$ and $j$ is then estimated as:

$$\hat{\rho}_{ij}=sign(\hat{\beta}_{j}^{(i)})\sqrt{\hat{\beta}_{j}^{(i)}\hat{\beta}_{i}^{(j)}}$$

It can be shown that Formulation 1 and Formulation 2 are equivalent in the sense that they always yield the same partial correlation estimates [[7](#_ENREF_7)].

**Methods based on formulation 1:**

**B1) Simple Partial Correlation**

As described in Formulation 1, this method relies on the inversion of the covariance (or correlation) matrix to obtain the concentration (or precision) matrix denoted with $W$. In cases where the number of observations is less than the number of features (n < p), covariance matrices might be singular with some zero eigenvalues. Singular matrices can also be inverted with pseudo-inverse approaches to obtain partial correlations.

**B2) GeneNet** [[8](#_ENREF_8)]**:**

This method first shrinks the covariance matrix towards a low-dimensional target value so that a well-conditioned and positive definite matrix is obtained. The shrunken covariance matrix is then inverted to obtain partial correlations. The shrinkage parameter $\lambda$ is chosen automatically from the data. We describe the shrinkage estimation steps in more detail below.

1. The Ledoit-Wolf lemma: An analytic approach for determining the optimal shrinkage level

Ledoit and Wolf [[9](#_ENREF_9)] developed an analytic approach for determining the optimal shrinkage level in large-dimensional estimation problems.

Let $\boldsymbol{\Psi}=(\psi_{1},\ldots,\psi_{p})$ denote the parameters of the unrestricted high-dimensional model of interest, and $\boldsymbol{\Theta}=(\theta_{i})$ the matching parameters of a lower dimensional restricted submodel.

For instance, $\boldsymbol{\Psi}$ could be the mean vector of a p-dimensional multivariate normal, and $\boldsymbol{\Theta}$ the vector of a corresponding constrained submodel where the means are all assumed to be equal (*i.e.* $\theta_{1}=\theta_{2}=\ldots=\theta_{p}$). By fitting each of the two different models to the observed data, we obtain the associated estimates $\boldsymbol{U=}\hat{\boldsymbol{\Psi}}$ and $\boldsymbol{T=}\hat{\boldsymbol{\Theta}}$. The unconstrained estimate $\boldsymbol{U}$ will exhibit a comparatively high variance due to the larger number of parameters that need to be fitted, whereas its low-dimensional counterpart $\boldsymbol{T}$ will have lower variance but potentially also considerable bias as an estimator of the true $\boldsymbol{\Psi}$.

Instead of choosing between one of these two extremes, the linear shrinkage approach suggests to combine both estimators in a weighted average

$$\boldsymbol{U}^{\boldsymbol{*}}=\lambda\boldsymbol{T}+(1-\lambda)\boldsymbol{U}$$

where $\lambda\in[0,1]$ denotes the shrinkage intensity. This is a convex combination of $\boldsymbol{T}$ and $\boldsymbol{U}$, and by setting $\lambda$ equal to 1 or 0, it is possible to recover the original estimates $\boldsymbol{T}$ and $\boldsymbol{U}$ respectively. However, the regularized estimate $\boldsymbol{U}^{\boldsymbol{*}}$ outperforms both $\boldsymbol{T}$ and $\boldsymbol{U}$in terms of accuracy and statistical efficiency.

A **key question** in this procedure is how to select an optimal value for the shrinkage parameter $\lambda$. A systematic way is to choose it in a data-driven fashion by explicitly minimizing a risk function such as mean squared error (MSE):

$$R\left( \lambda\right)=E\left( L\left( \lambda\right) \right)=E(\sum_{i=1}^{p} {(u_{i}^{*}-\psi_{i})}^{2})$$

Ledoit and Wolf [[9](#_ENREF_9)] derived a closed-form solution for choosing $\lambda$ that guarantees minimal MSE without the need of having to specify the underlying distributions, and without requiring computationally expensive procedures such as MCMC, the bootstrap, or cross-validation.

$$\lambda^{*}=\frac{\sum_{i=1}^{p} Var(u_{i})-Cov\left( u_{i},t_{i} \right)-Bias\left( u_{i} \right)E({t_{i}-u}_{i})}{\sum_{i=1}^{p} E[{(t_{i}-u_{i})}^{2}]}$$

which is (apart from some algebraic simplification) the expression given in Ledoit and Wolf [[9](#_ENREF_9)].

1. Estimation of the optimal shinkage intensity

Schafer and Strimmer [[8](#_ENREF_8)] proposed to compute $\lambda^{*}$ by replacing all expectations, variances, and covariances in the above equation by their *unbiased* sample estimators. This leads to:

$$\hat{\lambda^{*}}=\frac{\sum_{i=1}^{p} \hat{Var}(u_{i})-\hat{Cov}\left( u_{i},t_{i} \right)-\hat{Bias}\left( u_{i} \right)E({t_{i}-u}_{i})}{\sum_{i=1}^{p} E[{(t_{i}-u_{i})}^{2}]}$$

In finite samples, $\lambda^{*}$ may exceed the value one, and in some cases it may even become negative. In order to avoid over-shrinkage or negative shrinkage, we truncate the estimated intensity correspondingly, using $\lambda^{**}=max(0,\min\left( 1,\lambda^{*} \right))$ when constructing the shrinkage estimator.

1. Shrinkage estimation of the covariance matrix

In a matrix setting, the equivalent to the squared error loss function is the squared Frobenius norm. Thus,

$$L\left( \lambda\right)=\left\| S^{*}-\Sigma\right\|_{F}^{2}$$

$=\left\| \lambda T+\left( 1-\lambda\right)S-\Sigma\right\|_{F}^{2}$

$=\sum_{i=1}^{p} \sum_{j=1}^{p} {(\lambda t_{ij}+\left( 1-\lambda\right)s_{ij}-\sigma_{ij})}^{2}$

is a natural quadratic measure of distance between the true $\Sigma$ and the inferred covariance matrix $S^{*}$. In this formula, the unconstrained empirical covariance matrix $S$ replaces the unconstrained estimate $\boldsymbol{U}$**.** In this setting, $\lambda^{*}$takes the form:

$$\hat{\lambda^{*}}=\frac{\sum_{i=1}^{p} \sum_{j=1}^{p} \hat{Var}(s_{ij})-\hat{Cov}\left( s_{ij},t_{ij} \right)-\hat{Bias}\left( s_{ij} \right)({t_{ij}-s}_{ij})}{\sum_{i=1}^{p} \sum_{j=1}^{p} {(t_{ij}-s_{ij})}^{2}}$$

1. The choice of a target matrix $\boldsymbol{T}$

In general, the choice of a target matrix should be guided by the presumed lower dimensional structure in the dataset as this determines the increase of efficiency over that of the empirical covariance. However, it is also a remarkable consequence of the above $\lambda^{*}$ formulation that, in fact, *any* target will lead to a reduction in MSE, albeit only a minor reduction in case of a strongly misspecified target (in which case $S^{*}$ will simply reduce to the unconstrained estimate $S$).

In GeneNet, Schafer and Strimmer [[8](#_ENREF_8)] implemented a shrinkage target that shrinks off-diagonal entries to zero, but leaves diagonal entries intact. This is called the “diagonal, unequal variance” model, and has *p* estimated parameters (the variances $s_{ii}$).

$$t_{ij}=\left\{ \begin{aligned} s_{ii} if i=j \\ 0 if i\neq j \end{aligned} \right.$$

1. The formulae for the shrunk covariances and correlations

With the choice of $\boldsymbol{T}$ above, $\hat{Cov}\left( s_{ij},t_{ij} \right)$ can take one of two values:

$$\hat{Cov}\left( s_{ij},t_{ij} \right)=\left\{ \begin{aligned} \hat{{Var(s}_{ii})} if i=j \\ 0 if i\neq j \end{aligned} \right.$$

$\hat{Bias}\left( s_{ij} \right)({t_{ij}-s}_{ij})$, on the other hand, disappears from the equation if we use the unbiased sample estimates for $s_{ij}$. So, for this choice of $\boldsymbol{T}$ and $s_{ij}$, the closed-form solution of $\lambda^{*}$ becomes:

$$\hat{\lambda^{*}}=\frac{\sum_{i\neq j} \hat{Var(s_{ij})}}{\sum_{i\neq j} s_{ij}^{2}}$$

We can also choose to shrink the correlations instead of the variances. In this case, the formula takes the form:

$$\hat{\lambda^{*}}=\frac{\sum_{i\neq j} \hat{Var(r_{ij})}}{\sum_{i\neq j} r_{ij}^{2}}$$

The shrunk correlation and covariance estimates $r_{ij}^{*}$ and $s_{ij}^{*}$ are then given by:

$$r_{ij}^{*}=\left\{ \begin{aligned} s_{ii} if i=j \\ r_{ij}^{*} max(0,\min\left( 1,\lambda^{*} \right)) if i\neq j \end{aligned} \right.$$

$$s_{ij}^{*}=\left\{ \begin{aligned} s_{ii} if i=j \\ r_{ij}^{*}\sqrt{s_{ii}s_{jj}} if i\neq j \end{aligned} \right.$$

**B3) Graphical lasso (GLASSO):**

The graphical lasso [[10](#_ENREF_10)] is an algorithm that learns the structure of an undirected Gaussian graphical model using an $\mathcal{l}_{1}$ regularization to control the number of zeros in the precision matrix $\Theta=\Sigma^{-1}$.

In this algorithm, the n observations in the design matrix $X$ are assumed to be multivariate normal with mean $\mu$ and covariance $\Sigma$ . Let $S$ be the empirical covariance matrix, then the Gaussian log-likelihood of the data, partially maximized with respect to the mean parameter $\mu$ is

$$\log\det\Theta-tr\left( S\Theta\right)-\rho\left\| \Theta\right\|_{1}$$

Here $tr$ denotes the trace and $\left\| \Theta\right\|_{1}$ is the $\mathcal{l}_{1}$ norm - the sum of the absolute values of the elements of $\Sigma^{-1}$. GLASSO uses a block-coordinate descent method to maximize this log-likelihood over non-negative definite matrices $\Theta$ and achieves fast convergence.

**Methods based on formulation 2:**

**B4) Partial least squares (PLS) regression:** PLS is a method for supervised dimensionality reduction. The main idea is to build a few orthogonal components from $X^{(\backslash i)}$ (all features but the i^th^ one), and to use them as predictors in a least squares fit for $X^{(i)}$ [[6](#_ENREF_6)]. In this fashion, a PLS-based regression model is computed for each protein in the dataset.

A PLS component $t=X^{(\backslash i)}w$ is a linear combination of the original predictors that have maximal covariance with the response vector $X^{(i)}$ with the additional stipulation that the components are mutually orthogonal. The weights for the k^th^ PLS component are found as:

$$w_{k}=arg\max_{\left\| w \right\|=1} cov{(X^{\left( \backslash i \right)}w,X^{(i)})}^{2}$$

s.t. $w_{k}^{T} X^{\left( \backslash i \right) T} X^{\left( \backslash i \right)} w_{l}=0$ for $l<k$

The maximum number of components that can be used is specified by the user. In this study, the optimal number for each regression model is determined via k-fold cross-validation.

PLS regression is particularly suited when the matrix of predictors has more features than observations ($p>n$), and when there is multicollinearity among the features. By contrast, standard regression will fail in these cases unless regularized [[11](#_ENREF_11)].

**B5) Ridge regression:** This method involves adding an $\mathcal{l}_{2}$ penalty term $\lambda\left\| \boldsymbol{\beta} \right\|_{2}^{2}$to the least squares crtierion where $\lambda>0$ is a regularization parameter.

$${\hat{\boldsymbol{\beta}}}^{\boldsymbol{(i)}}=\arg\min_{\boldsymbol{\beta}\in\mathbb{R}^{p-1}} \left\| X^{(i)}-X^{(\backslash i)}\boldsymbol{\beta} \right\|^{2}+\lambda\left\| \boldsymbol{\beta} \right\|_{2}^{2}$$

A ridge regression model is computed for each protein in the dataset and partial correlation $\hat{\rho}_{ij}$is computed using regression coefficients $\hat{\beta}_{j}^{(i)}$ and $\hat{\beta}_{i}^{(j)}$from the models for protein $i$ and $j$. In this study, the optimal value of $\lambda$ is determined via k-fold cross-validation.

**B6) Lasso regression:** Similar to ridge regression, the estimated regression coefficients are chosen to optimize a penalized least squares criterion. Lasso regression is based on an $\mathcal{l}_{1}$-penalty $\lambda\left\| \boldsymbol{\beta} \right\|_{1}$ where $\lambda>0$ is the regularization parameter. The least squares criterion then takes the form

$${\hat{\boldsymbol{\beta}}}^{\boldsymbol{(i)}}=\arg\min_{\boldsymbol{\beta}\in\mathbb{R}^{p-1}} \left\| X^{(i)}-X^{(\backslash i)}\boldsymbol{\beta} \right\|^{2}+\lambda\left\| \boldsymbol{\beta} \right\|_{1}$$

A matrix of partial correlations is estimated based on a lasso regression model for each of the columns (proteins) in the data matrix. The sparsity parameter $\lambda$ can be estimated using k-fold cross-validation, which results in the oracle penalty for optimal prediction. However, in this study we used a $\lambda$ value that is common for all regression models in a network and maximizes area under the precision-recall curve (AUPR).

Lasso is not the ideal method in cases where the number of features is much higher than the number of observations (p ≫ n), or where there are highly correlated features [[12](#_ENREF_12)]. This is because lasso can only select at most n variables out of p candidates; and it tends to select one variable from a group of highly correlated features and ignore the others.

**B7) Elastic Net:** This method is a regularized regression method that linearly combines the $\mathcal{l}_{1}$ and $\mathcal{l}_{2}$ penalties of the lasso and ridge methods. The estimates from elastic net when fitting a model for ${\hat{\boldsymbol{\beta}}}^{\boldsymbol{(i)}}$ are defined by:

$${\hat{\boldsymbol{\beta}}}^{\boldsymbol{(i)}}=\arg\min_{\boldsymbol{\beta}\in\mathbb{R}^{p-1}} \left\| X^{(i)}-X^{(\backslash i)}\boldsymbol{\beta} \right\|^{2}+\lambda_{1}\left\| \boldsymbol{\beta} \right\|_{1}+\lambda_{2}\left\| \boldsymbol{\beta} \right\|_{2}^{2}$$

The naive version of elastic net finds an estimator in a two-stage procedure: it first finds ridge regression coefficients for each fixed $\lambda_{1}$, and then performs a lasso type shrinkage. This kind of estimation leads to increased bias and poor predictions due to the double amount of shrinkage. To alleviate this problem, Friedman et al. (2010) propose to fit the model using:

$${\hat{\boldsymbol{\beta}}}^{\boldsymbol{(i)}}=\arg\min_{\boldsymbol{\beta}\in\mathbb{R}^{p-1}} \left\| X^{(i)}-X^{(\backslash i)}\boldsymbol{\beta} \right\|^{2}+\alpha\left\| \boldsymbol{\beta} \right\|_{1}+{\frac{1-\alpha}{2}\left\| \boldsymbol{\beta} \right\|}_{2}^{2}$$

Elastic net encourages the grouping effect and thus can have improved prediction power compared with lasso. The grouping effect is the situation where the regression coefficients of a group of highly correlated variables tend to be equal (up to a sign change if negatively correlated) in a regression model [[12](#_ENREF_12)].In particular, the regression method should assign identical coefficients to identical variables if there are any.

**C) Methods based on mutual information:**

The mutual information between protein $i$ and protein $j$ is denoted with $MI\left( X_{i};X_{j} \right)$ and abbreviated below with $MI\left( i;j \right)$ if both components are a feature in $X$. It can be efficiently computed using entropy estimates from K-nearest neighbor distances [[5](#_ENREF_5)].

**C1) ARACNE additive (ARACNE-A):** ARACNE-A takes the mutual information matrix of proteins as input, considers each triple of edges independently, and removes the weakest edge $i\leftrightarrow j$ if

$MI\left( i;j \right)<MI\left( j;k \right)-\varepsilon$ and $MI\left( i;j \right)<MI\left( i;k \right)-\varepsilon$

are both true [[13](#_ENREF_13)]. The output is a weighted adjacency matrix of the inferred network. $\varepsilon$ is a user-specified parameter.

**C2) ARACNE multiplicative (ARACNE-M):** This method works in a way similar to ARACNE-A, but with the difference that the decision criterion to remove the weakest edge involves a multiplicative threshold as opposed to an additive one. ARACNE-M removes the weakest edge $i\leftrightarrow j$ if

$MI\left( i;j \right)<MI\left( j;k \right)*(1-\tau)$ and $MI\left( i;j \right)<MI\left( i;k \right)*(1-\tau)$

are both satisfied. $\tau$ is a user-specified parameter.

**C3) Context-likelihood of relatedness (CLR):** This method computes the score $\sqrt{z_{i}^{2}+z_{j}^{2}}$ for each pair of variables $i$ and $j$ where

$$z_{i}=max(0,\frac{MI\left( i;j \right)-\mu_{i}}{\sigma_{i}})$$

and, $\mu_{i}$ and $\sigma_{i}$ are the mean and standard deviation of the mutual information values for all $MI\left( i;k \right)$ such that k = 1,...,n [[14](#_ENREF_14)]. Given the mutual information matrix, CLR computes the weighted adjacency matrix without a need for any internal parameters.

**C4) MRNET:** This method uses the mutual information between data objects (i.e proteins) and a feature selection algorithm [minimum-redundancy-maximum-relevance (MRMR)] to infer interactions between objects [[15](#_ENREF_15)].It first starts by selecting the variable $X_{i}$ having the highest mutual information with the target $Y$. Then it repeatedly enlarges the set of selected variables $S$ by taking the $X_{k}$ that maximizes $MI\left( X_{k};Y \right)-mean(MI\left( X_{k};X_{i} \right))$ for all $X_{i}$ already in $S$. The procedure stops when the score becomes negative.

**REFERENCES**

1. Grote T, Siwak DR, Fritsche HA, Joy C, Mills GB, et al. (2008) Validation of reverse phase protein array for practical screening of potential biomarkers in serum and plasma: Accurate detection of CA19-9 levels in pancreatic cancer. Proteomics 8: 3051-3060.

2. Tibes R, Qiu YH, Hennessy B, Andreeff M, Miiis GB, et al. (2006) Reverse phase protein array: validation of a novel proteomic technology and utility for analysis of primary leukemia specimens and hematopoietic stem cells. Mol Cancer Ther 5: 2512-2521.

3. Marbach D, Costello JC, Kuffner R, Vega NM, Prill RJ, et al. (2012) Wisdom of crowds for robust gene network inference. Nat Methods 9: 796-+.

4. Girvan M, Newman MEJ (2002) Community structure in social and biological networks. P Natl Acad Sci USA 99: 7821-7826.

5. Kraskov A, Stogbauer H, Grassberger P (2004) Estimating mutual information. Physical review E, Statistical, nonlinear, and soft matter physics 69: 066138.

6. Kramer N, Schafer J, Boulesteix AL (2009) Regularized estimation of large-scale gene association networks using graphical Gaussian models. Bmc Bioinformatics 10: 384.

7. Whittaker J (1990) Graphical Models in Applied Multivariate Statistics. New York: Wiley.

8. Schafer J, Strimmer K (2005) A shrinkage approach to large-scale covariance matrix estimation and implications for functional genomics. Stat Appl Genet Mo B 4.

9. Ledoit O, Wolf M (2003) Improved estimation of the covariance matrix of stock returns with an application to portfolio selection. Journal of Empirical Finance 10: 603-621.

10. Friedman J, Hastie T, Tibshirani R (2008) Sparse inverse covariance estimation with the graphical lasso. Biostatistics 9: 432-441.

11. Wikipedia PLSR Partial least squares regression.

12. Zou H, Hastie T (2005) Regularization and variable selection via the elastic net. Journal of the Royal Statistical Society: Series B (Statistical Methodology) 67: 301-320.

13. Margolin AA, Nemenman I, Basso K, Wiggins C, Stolovitzky G, et al. (2006) ARACNE: An algorithm for the reconstruction of gene regulatory networks in a mammalian cellular context. Bmc Bioinformatics 7.

14. Faith JJ, Hayete B, Thaden JT, Mogno I, Wierzbowski J, et al. (2007) Large-scale mapping and validation of Escherichia coli transcriptional regulation from a compendium of expression profiles. Plos Biol 5: 54-66.

15. Peng HC, Long FH, Ding C (2005) Feature selection based on mutual information: Criteria of max-dependency, max-relevance, and min-redundancy. Ieee T Pattern Anal 27: 1226-1238.
