# Supplementary figures and images for: A Multi-Method Approach for Proteomic Network Inference in 11 Human Cancers
Source: PLoS Comput Biol. 2016 Feb 29;12(2):e1004765. doi: 10.1371/journal.pcbi.1004765 (PMC4771175; doi:10.1371/journal.pcbi.1004765)

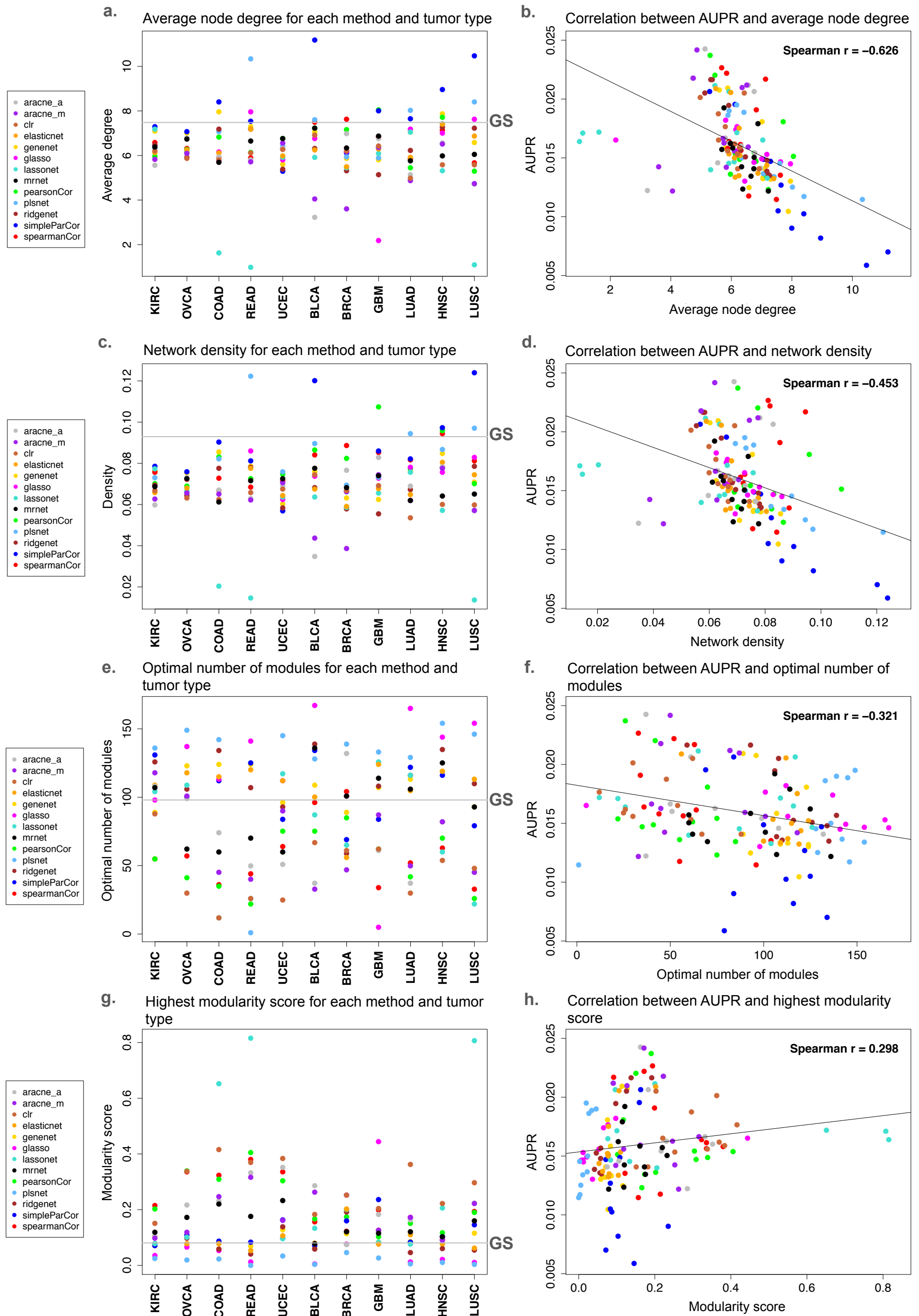

Supplement: S1 Fig — (A,C,E,G) The x-axis shows tumor types while the y-axis shows (A) average node degree, (C) network density, (E) optimal number of modules, and (G) highest modularity score. Colors denote different methods as shown in the legend. (B,D,F,H) Scatter plots of these network statistics with AUPR. (PDF) [file pcbi.1004765.s002.pdf]

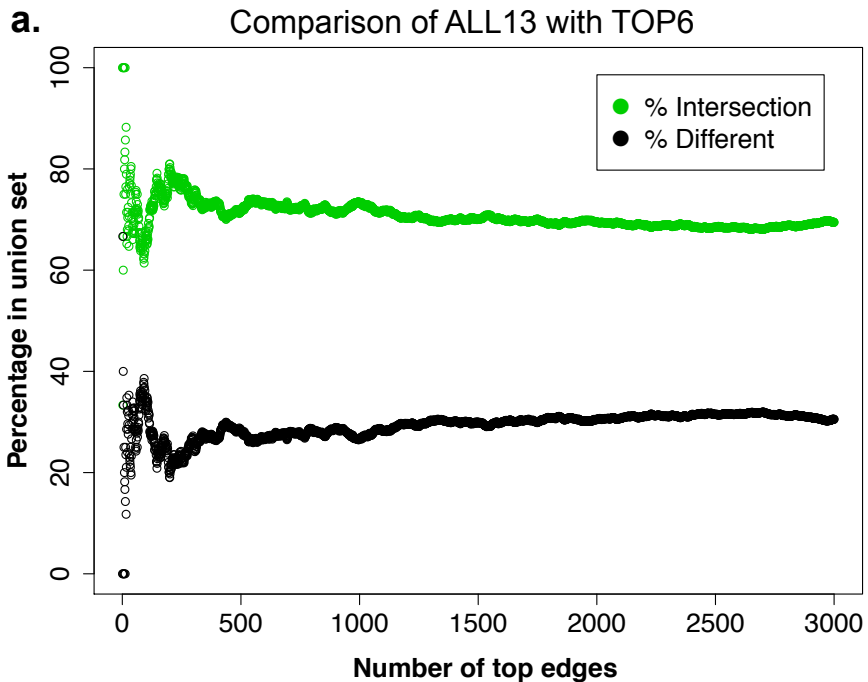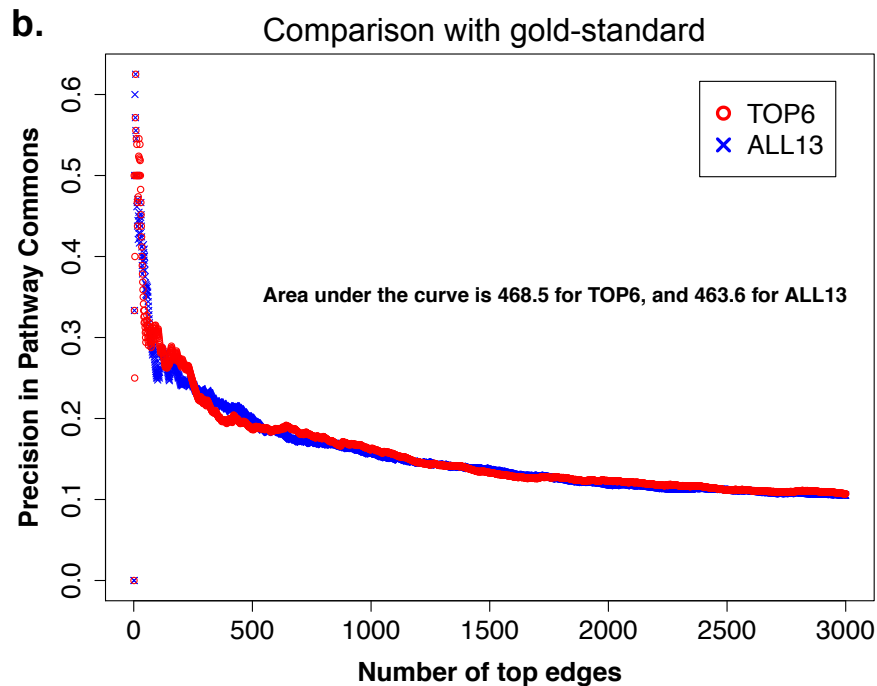

Supplement: S3 Fig — First, a pan-cancer rank for each edge is computed separately from the TOP6 and the ALL13 methods. Edge lists ordered according to these pan-cancer ranks are then compared. (A) Overlap and difference percentage between the significant ALL13 and TOP6 edges as the significance threshold is varied from 1 to 3000. (B) Precision of the significant ALL13 and TOP6 edges in the gold-standard network as the significance threshold is varied from 1 to 3000. (PDF) [file pcbi.1004765.s004.pdf]

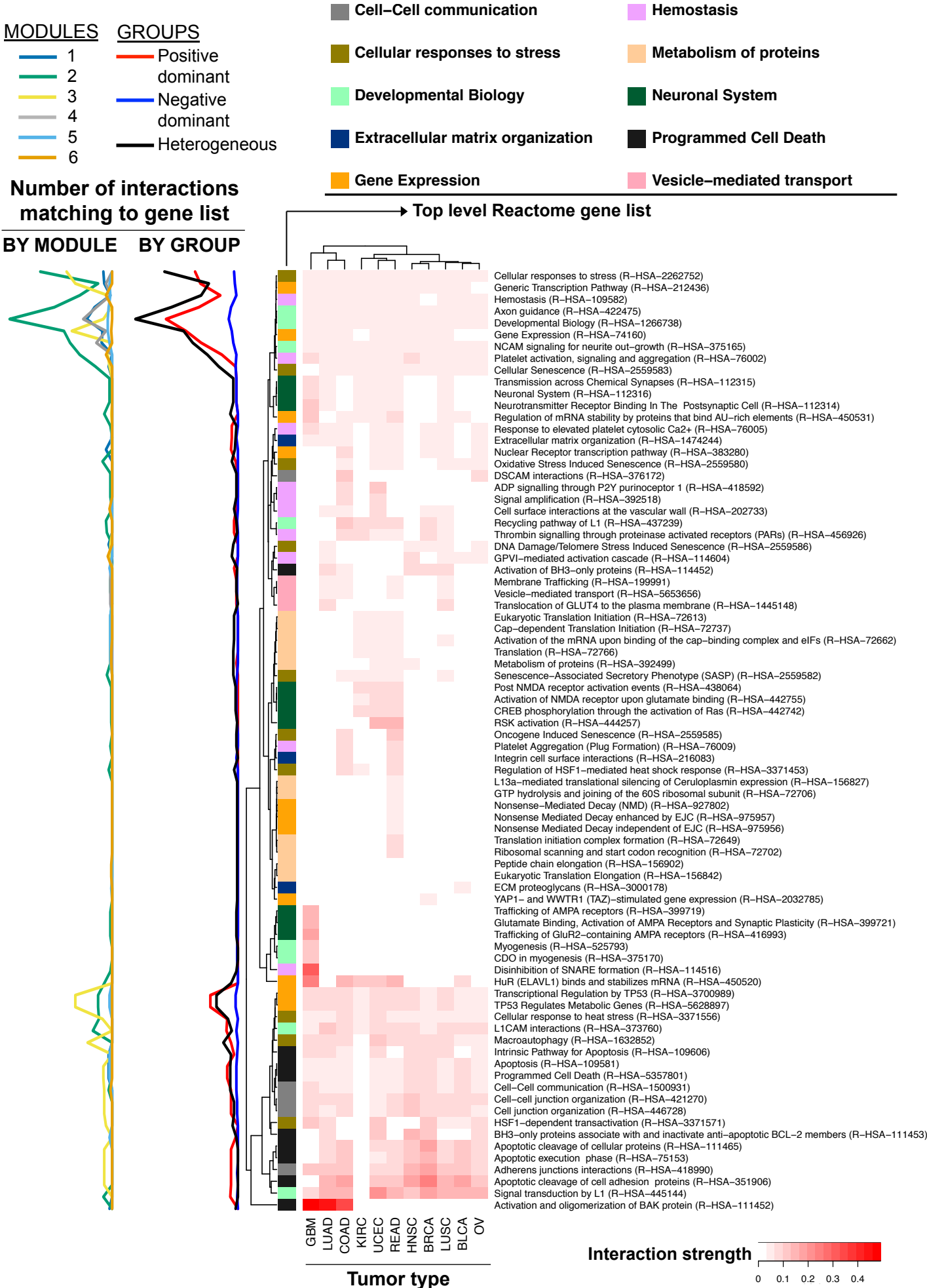

Supplement: S8 Fig — Per tumor type average interaction strengths for ten Reactome top-level events are shown. The number of gene lists for each top-level event is provided in Fig 8A. The number of significant (consensus rank < 425) interactions that match to each gene list is broken down by module or group, averaged over tumor types, and tracked on the left of the heatmap. Heat map orders for gene lists (rows) and tumor types (columns) are both obtained from Ward-linkage Euclidean-distance hierarchical clustering. The numbers for the ‘module’ lines may be zero if the matching interactions are inter-module (linking two modules). (PDF) [file pcbi.1004765.s009.pdf]
