# Supplementary material for: A Multi-Method Approach for Proteomic Network Inference in 11 Human Cancers
Source: PLoS Comput Biol. 2016 Feb 29;12(2):e1004765. doi: 10.1371/journal.pcbi.1004765 (PMC4771175; doi:10.1371/journal.pcbi.1004765)
Supplement: S2 Fig — In contrast with S1 Fig, methods are shown on the x-axis and colors denote different tumor types. The y-axis shows (A) average node degree, (B) network density, (C) optimal number of modules, and (D) highest modularity score. (PDF) [file pcbi.1004765.s003.pdf]

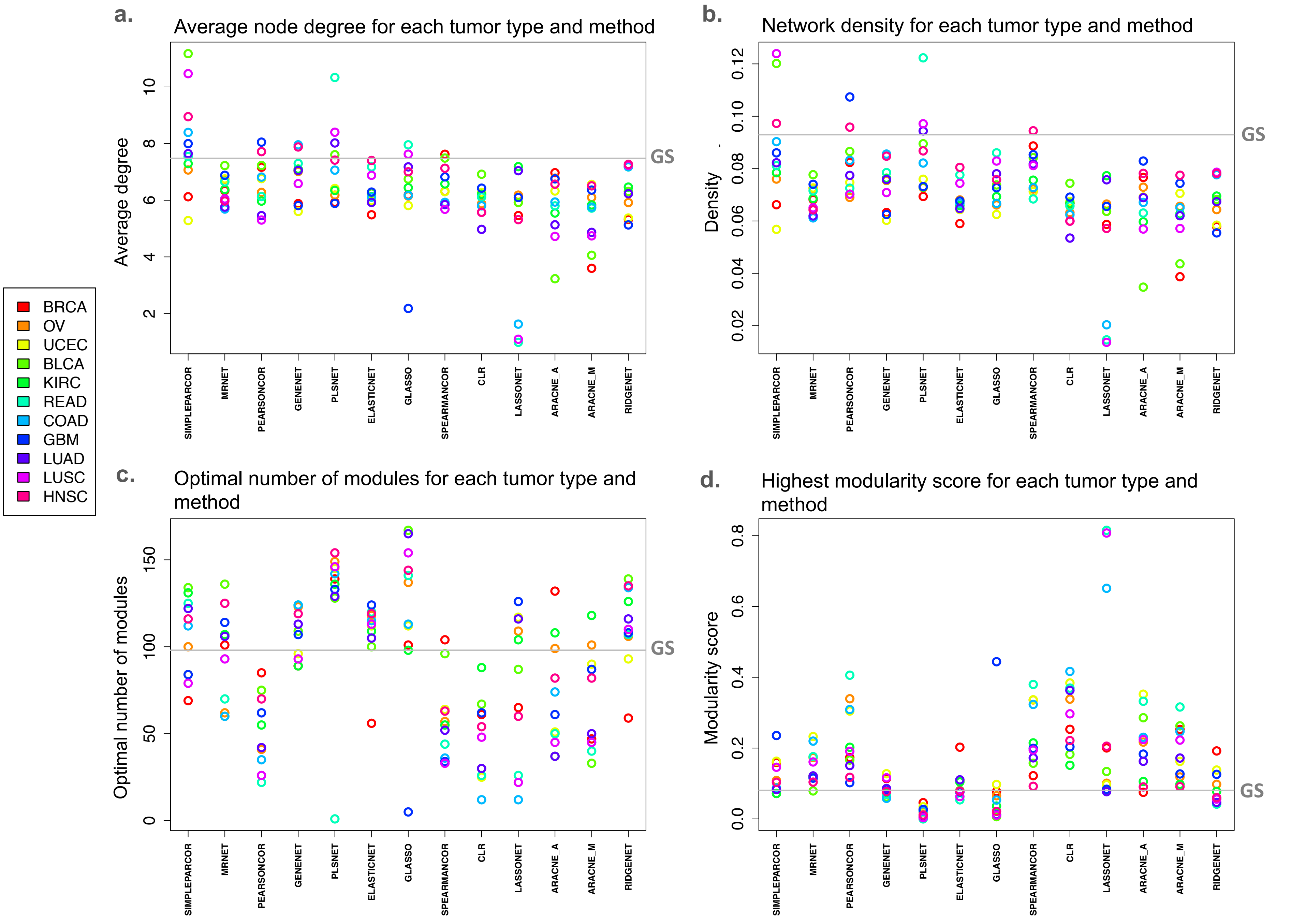

GS: Gold-standard from Pathway Commons
