# Supplementary material for: A Multi-Method Approach for Proteomic Network Inference in 11 Human Cancers
Source: PLoS Comput Biol. 2016 Feb 29;12(2):e1004765. doi: 10.1371/journal.pcbi.1004765 (PMC4771175; doi:10.1371/journal.pcbi.1004765)

a. Overlap percentage among tumors, and between tumors and a random network as the edge-rank-cutoff is increased from 25 to 2000.

b. Variance explained by the first 3 PCs as the edge-rank-cutoff is increased from 25 to 2000.

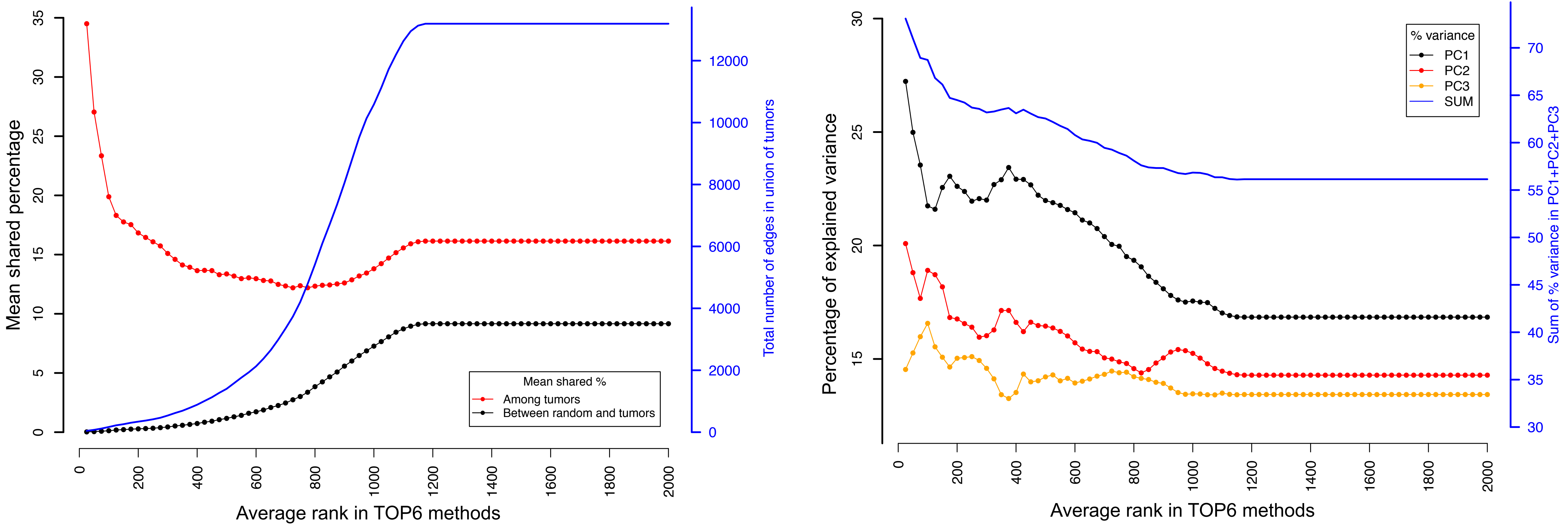

Supplement: S4 Fig — (A) The left y-axis shows the mean of the percentages of shared edges. The red dotted line is for the mean from all pairwise tumor-tumor comparisons. The black dotted line is for the mean from the 11 tumor-random comparisons each of which involve one distinct tumor type and the same randomly ordered edge list. The right y-axis shows the number of edges in the union set from all tumors (blue line). The x-axis shows the cutoff value for the TOP6 consensus edge ranks. (B) The left y-axis shows the percentage of variance explained by PC1, PC2 and PC3 (black, red, and orange dotted lines respectively). The right y-axis indicates the sum of the variance percentages explained by the first three principal components (blue line). The x-axis shows the cutoff value for the TOP6 consensus edge ranks. (PDF) [file pcbi.1004765.s005.pdf]
