# Supplementary material for: A Multi-Method Approach for Proteomic Network Inference in 11 Human Cancers
Source: PLoS Comput Biol. 2016 Feb 29;12(2):e1004765. doi: 10.1371/journal.pcbi.1004765 (PMC4771175; doi:10.1371/journal.pcbi.1004765)
Supplement: S5 Fig — (PDF) [file pcbi.1004765.s006.pdf]

	ARACNE_A		ARACNE_M		CLR	ELASTICNET		GLASSO	LASSONET	MRNET	PLSNET	RIDGENET
	Knn	epsilon	Knn	tau	Knn	K-fold	alpha	lambda	lambda	Knn	K-fold	K-fold
**BLCA**	6	0.0001	6	0.05556	6	5	0.55444	0.0604	0.08054	6	3	5
**BRCA**	4	0.06869	4	0.06566	4	5	0.33667	0.00671	0.00671	4	4	5
**COAD**	6	0.06869	6	0.31313	6	5	0.33667	0.01342	0.28188	6	10	5
**GBM**	6	0.23333	6	0.34848	4	4	0.55444	0.24832	0.02013	4	5	3
**HNSC**	4	0.20833	4	0.51042	4	5	0.33667	0.03356	0.12081	4	10	5
**KIRC**	2	0.09495	2	0.36364	3	5	0.44556	0.01342	0.00671	6	20	20
**LUAD**	6	0.1697	4	0.4899	6	4	0.44556	0.04027	0.07383	6	5	4
**LUSC**	5	0.21667	5	0.625	6	4	0.44556	0.02013	0.34228	6	3	4
**OV**	4	0.29167	6	0.18182	5	4	0.44556	0.00671	0.01342	4	10	10
**READ**	5	0.12323	5	0.30808	5	5	0.44556	0.01342	0.4094	6	4	3
**UCEC**	5	0.13535	5	0.28788	6	5	0.33667	0.00671	0.00671	6	20	20
