# Supplementary material for: A Multi-Method Approach for Proteomic Network Inference in 11 Human Cancers
Source: PLoS Comput Biol. 2016 Feb 29;12(2):e1004765. doi: 10.1371/journal.pcbi.1004765 (PMC4771175; doi:10.1371/journal.pcbi.1004765)
Supplement: S6 Fig — The high-performing TOP6 group is shown in the top panel, and the other methods in the bottom panel. All 13 methods are used for the PCA of methods, but only the TOP6 methods are used for the PCA of tumor types. (PDF) [file pcbi.1004765.s007.pdf]

TOP6 methods	BLCA	BRCA	COAD	GBM	HNSC	KIRC	LUAD	LUSC	OVCA	READ	UCEC	UNION
ARACNE_A	1249	1276	1057	1108	1110	1038	770	789	1268	1073	1164	6747
ARACNE_M	1338	1261	1025	1094	1100	1091	769	792	1141	1059	1227	7129
CLR	1294	1161	1085	1202	1043	1162	930	1042	1099	1143	1173	7020
LASSONET	1107	1021	1334	1140	1172	1344	1317	1350	1156	1100	1108	8275
RIDGENET	1174	985	1336	954	1359	1209	1159	1338	1095	1328	994	7547
SPEARMANCOR	1341	1319	972	1099	1084	1151	842	801	1112	1018	1122	5826
TOP6 UNION	3141	2981	2604	2944	3042	3269	2780	2441	3162	2700	3014	

Other methods	BLCA	BRCA	COAD	GBM	HNSC	KIRC	LUAD	LUSC	OVCA	READ	UCEC	UNION
ELASTICNET	1163	1026	1124	1176	1370	1146	1090	1280	1158	1335	1119	7277
GENENET	1318	1100	1488	1087	1474	1328	1319	1232	1312	1365	1048	8380
GLASSO	1242	1157	1148	1198	1303	1205	1328	1411	1150	1480	1087	7054
MRNET	1351	1186	1064	1287	1117	1193	1077	1131	1261	1244	1263	7923
PEARSONCOR	1214	1254	1125	1216	1250	1021	775	806	1149	1042	1231	5614
PLSNET	1301	1020	1222	954	1276	1111	1372	1462	1057	1757	1091	6499
SIMPLEPARCOR	2090	1139	1570	1496	1656	1364	1430	1780	1322	1411	988	10573
ALL13 UNION	6959	4534	5460	5890	5882	4793	5155	6247	4653	6376	4253	
