# Supplementary material for: A Multi-Method Approach for Proteomic Network Inference in 11 Human Cancers
Source: PLoS Comput Biol. 2016 Feb 29;12(2):e1004765. doi: 10.1371/journal.pcbi.1004765 (PMC4771175; doi:10.1371/journal.pcbi.1004765)
Supplement: S7 Fig — Top-level Reactome gene lists are annotated on the network with connections to child gene lists. Figure adopted from Reactome Pathway Browser. (PDF) [file pcbi.1004765.s008.pdf]

REACTOME 3.0 54

Pathways for: **Homo sapiens**

Analysis: Tour: Layout:

Event Hierarchy:

- U Cell Cycle
- U Cell-Cell communication
- U Cellular responses to stress
- U Chromatin organization
- Circadian Clock
- Developmental Biology
- U Disease
- U DNA Repair
- DNA Replication
- U Extracellular matrix organization
- U Gene Expression
- U Hemostasis
- U Immune System
- N Mitophagy
- U Metabolism
- U Metabolism of proteins
- Muscle contraction
- Neuronal System
- Organelle biogenesis and maintenance
- U Programmed Cell Death
- Reproduction
- U Signal Transduction
- U Transmembrane transport of small mo
- U Vesicle-mediated transport

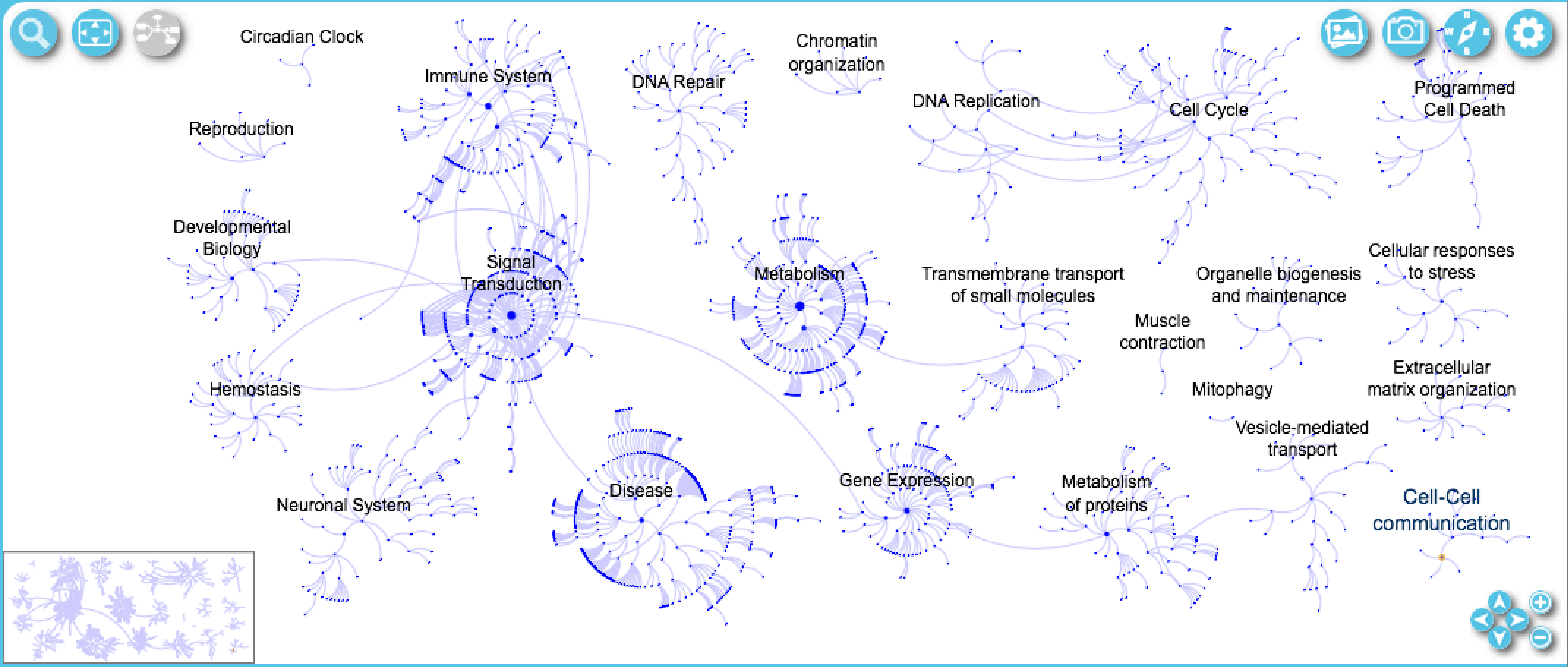

Description | **Molecules** | **Structures** | **Expression** | **Analysis** | **Downloads**

Displays details when you select an item in the Pathway Browser. For example, when a reaction is selected, shows details including the input and output molecules, summary and references containing supporting evidence. When relevant, shows details of the catalyst, regulators, preceding and following events.
