# Supplementary material for: A Multi-Method Approach for Proteomic Network Inference in 11 Human Cancers
Source: PLoS Comput Biol. 2016 Feb 29;12(2):e1004765. doi: 10.1371/journal.pcbi.1004765 (PMC4771175; doi:10.1371/journal.pcbi.1004765)
Supplement: S1 Protocol — (PDF) [file pcbi.1004765.s021.pdf]

Introduction

ProtNet is a graph visualization tool for Cancer Proteomic Networks.

Currently, there are 3 main visualization options: **Default**, **Custom**, and **Upload**. You can switch between the options via the tabbed panel on the right hand side.

Visualizing A Network Under The Default Tab

- Select a **Cancer Study** via the corresponding drop down menu.

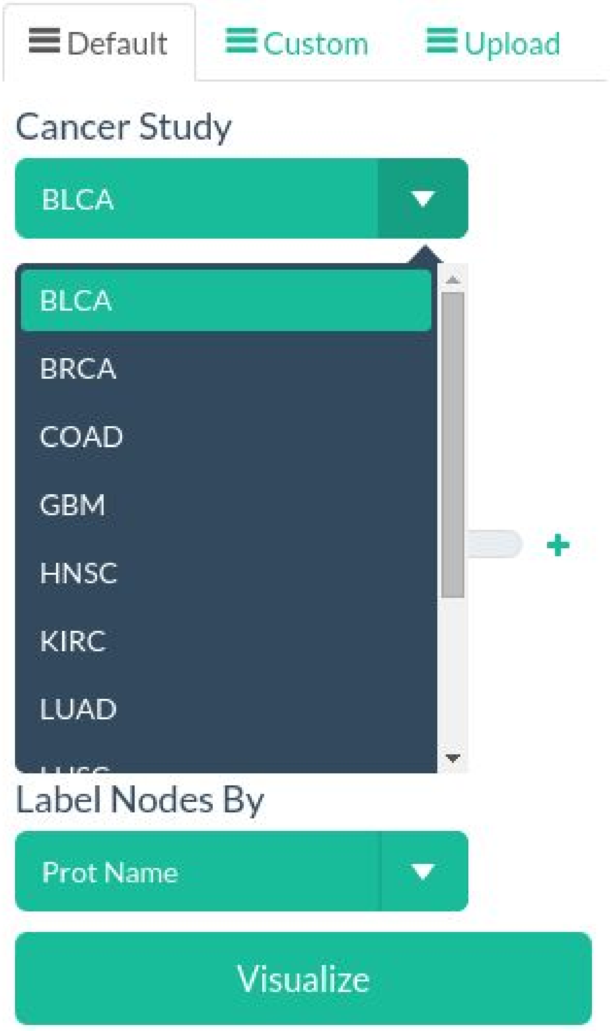

- Select a **Method**

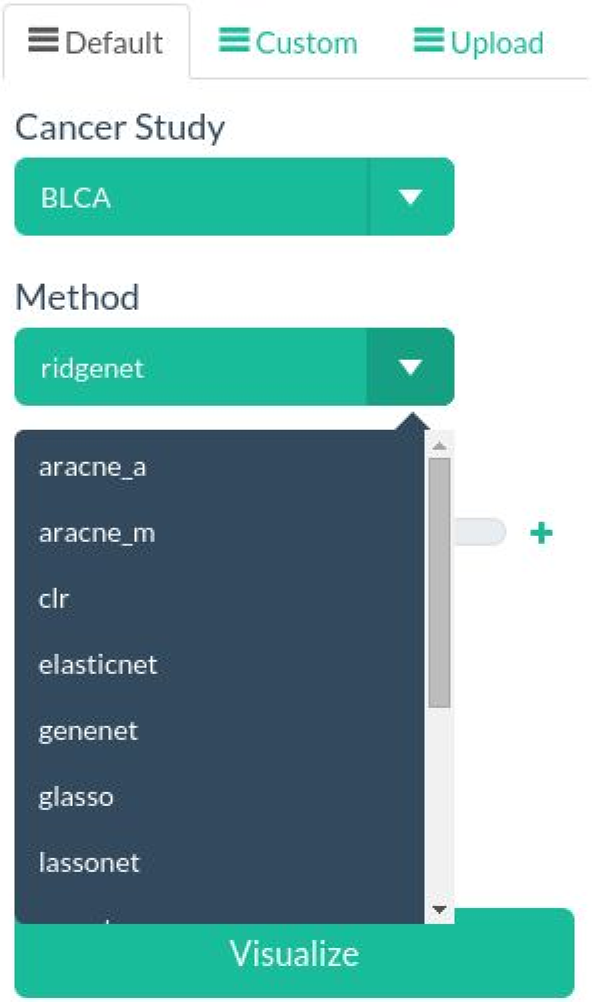

- Set the desired **Number of Edges** by using the slider.
- Press the **Visualize** button. This will generate a network for selected options.
- Once the visualization is complete, you can change edge coloring and node labels by using the corresponding drop-down menus.

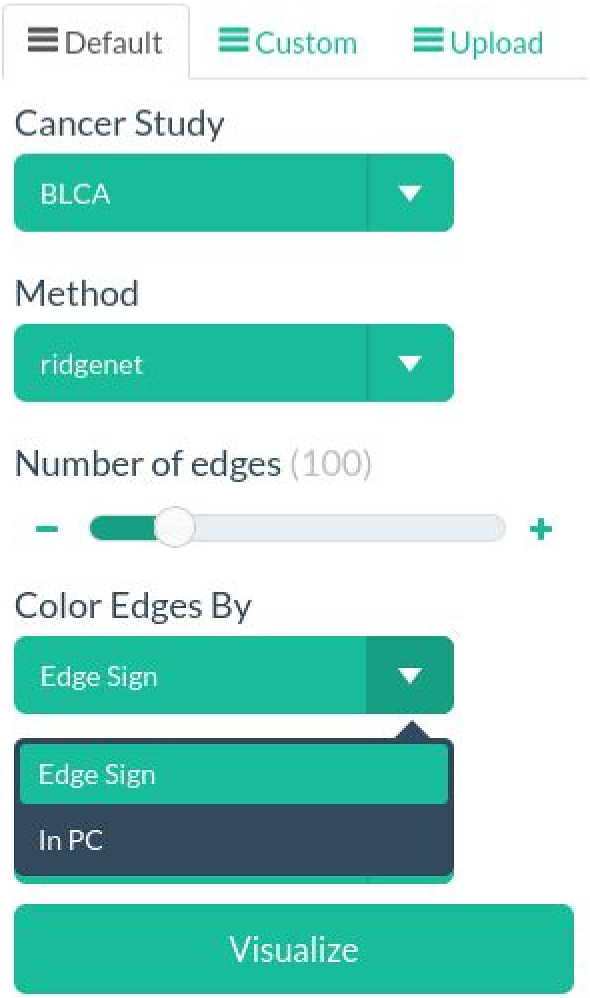

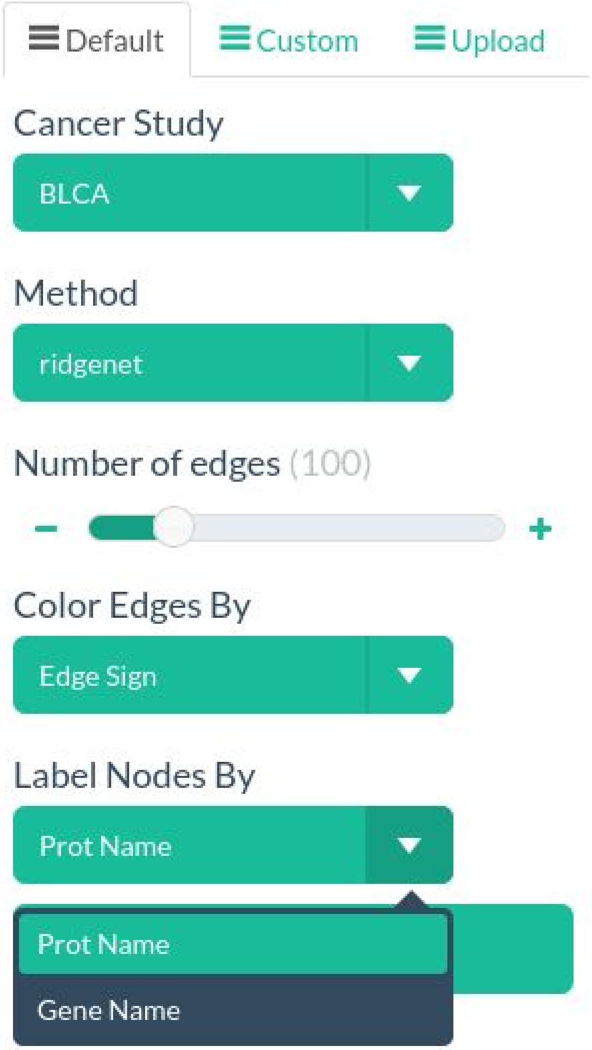

Visualizing A Network Under The Custom Tab

- First you have to specify a list of **Samples**. The easiest way to obtain a custom list of samples is to get it from the cBio Portal webpage.
    - Go to www.cbioportal.org
    - Select a cancer study, and then a patient/case set and enter a list of genes.

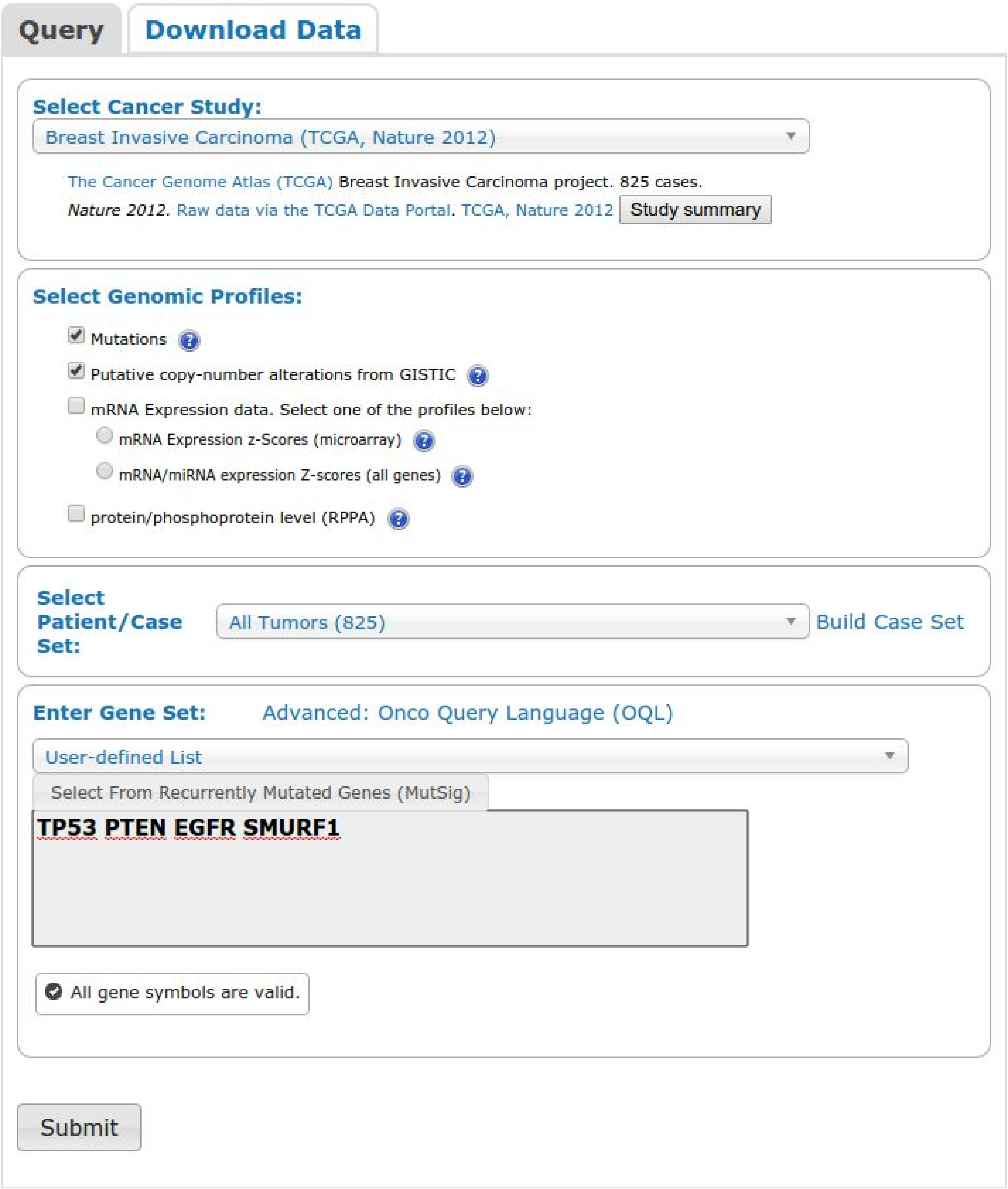

- After you submit the query you can find the list of samples under the download tab.

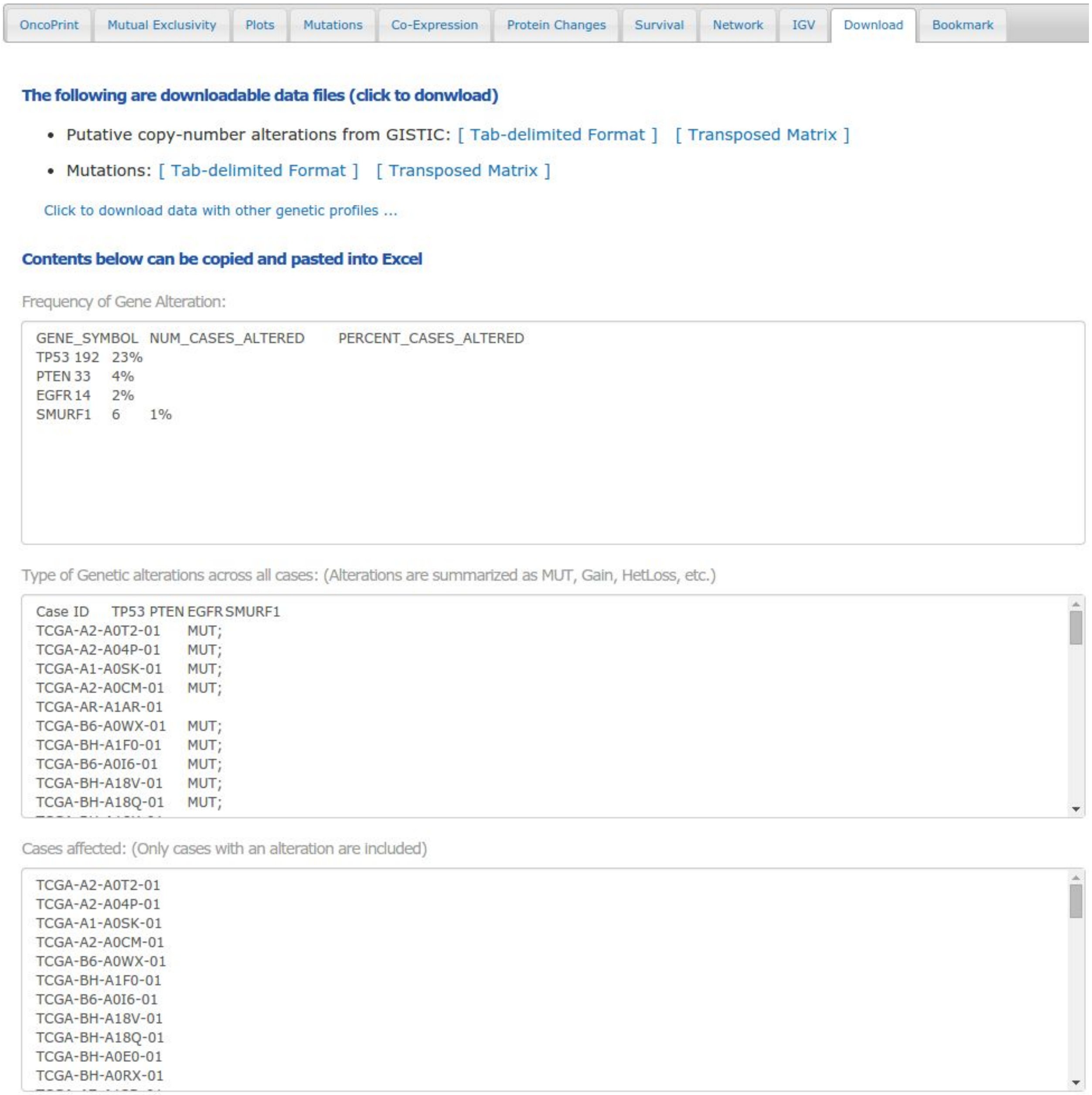

- ○ Alternatively you can query all cancer studies by just entering a list of genes. In this case, you may find the list of samples under mutations tab.

- Once you enter a list of samples, select a **Method** and set the **Number of Edges**.

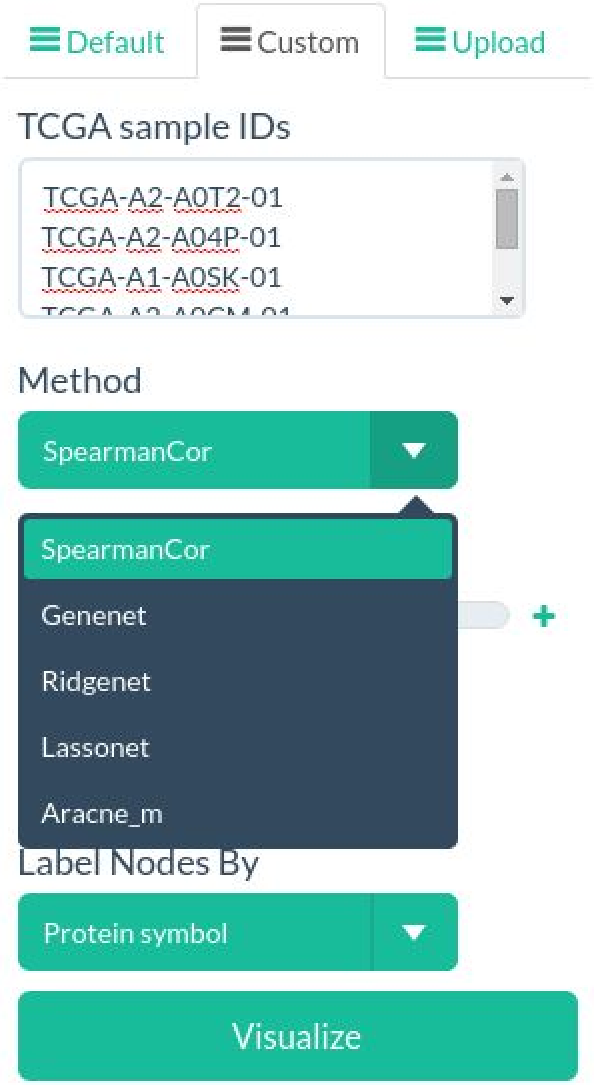

- Press the **Visualize** button. This will generate the custom network for the specified list of samples and the method.
- Similar to the default tab, you can change edge coloring and node labels by using the corresponding menus.

Visualizing A Network Under The Upload Tab

- First you have to select a tab-delimited data matrix file to upload. See **Data Matrix File Format** for details.

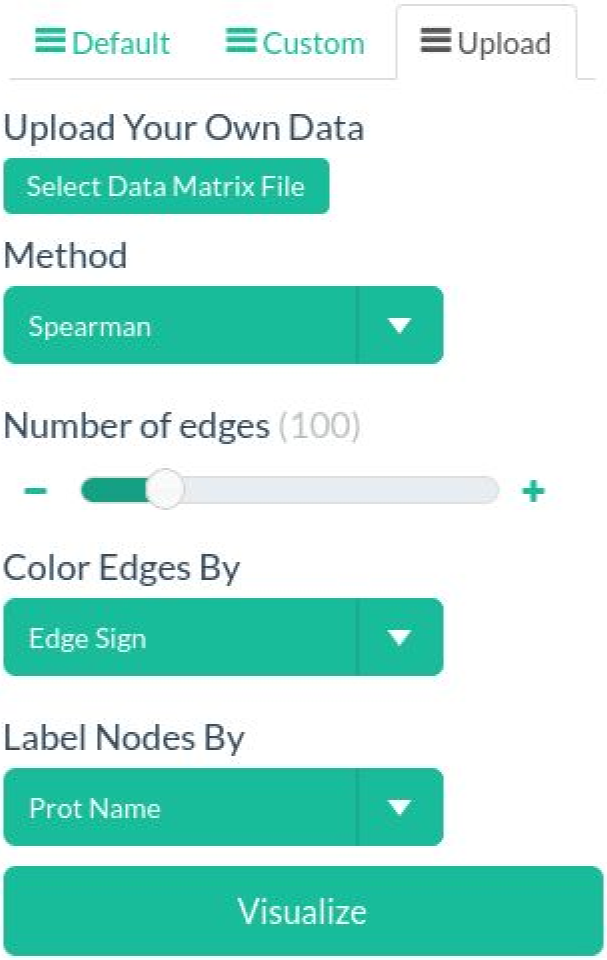

- Once you select the data matrix file to upload, select a **Method** and set the **Number of Edges**.
- Press the **Visualize** button. This will generate the network for the provided data matrix.

Performing Search On The Visible Network

You can search for a specific gene or protein within the visible network. Simply enter the gene or protein name into the search box located on top of the network view, and press the button or the enter key. Matching genes or proteins is highlighted with a different color.

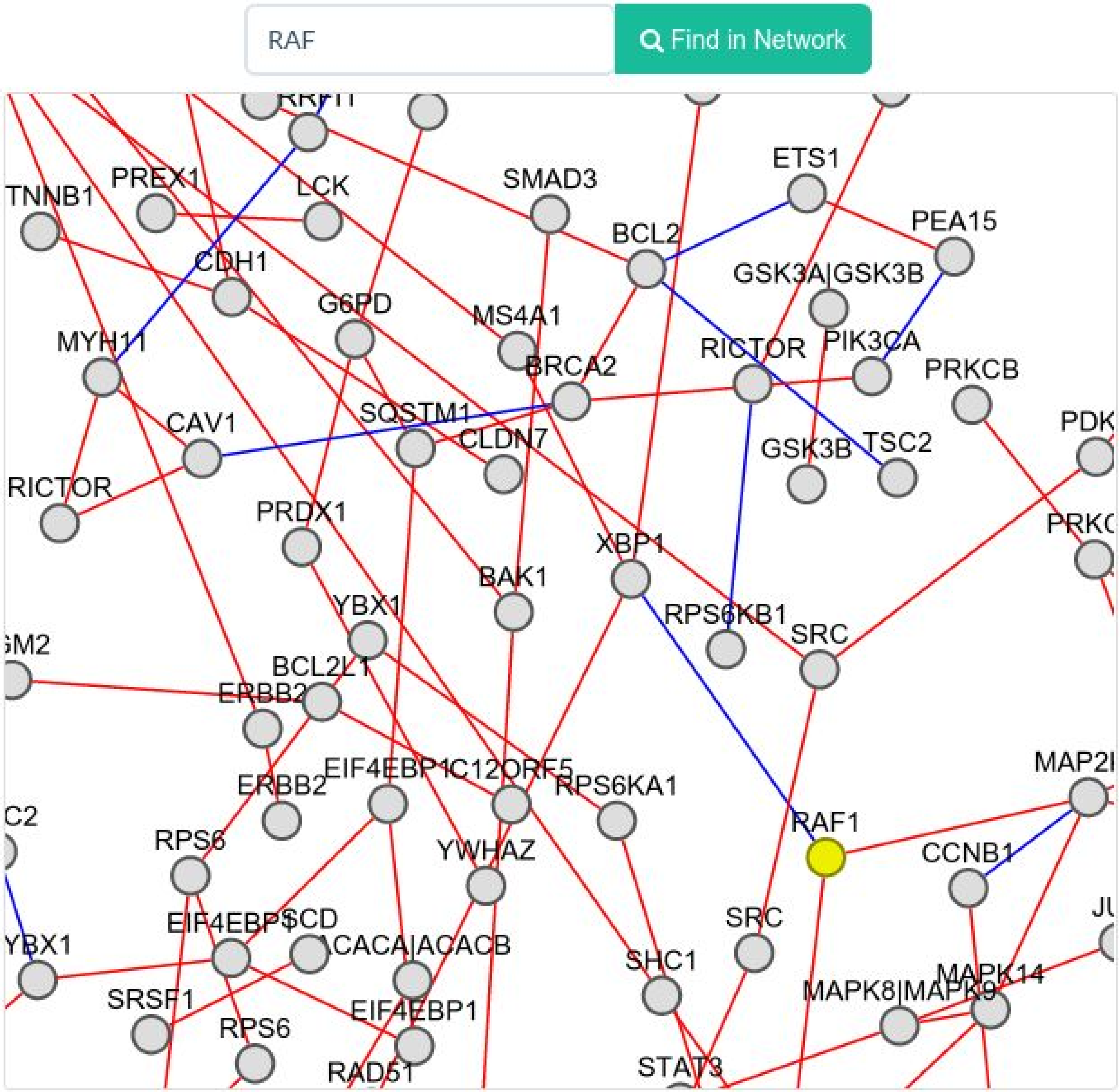

Data Matrix File Format

Currently, ProtNet allows users to upload their own data matrix files as tab delimited plain text files.

The first row of the file should be the gene/protein names. And, the first column should be sample IDs. Below you can find an example data matrix file in plain text format.

SampleID	Akt	Akt_pS473	Akt_pT308	AMPK_alpha	AMPK_pT172
TCGA-B6-A0I6	0.154298975	-0.35781673	-0.447332703	-0.574477553	-0.394586791
TCGA-BH-A0C1	-0.827756451	-1.038089747	0.967767764	-0.01783293	-0.16318257
TCGA-AR-A0U2	-0.799884711	-0.617498983	-0.068958312	-0.66862477	-1.990531056
TCGA-A2-A0YF	-0.974146829	-0.356275107	0.233013204	-0.462506111	-0.966453931
TCGA-BH-A18J	0.186297412	-0.482782305	0.354039455	0.116103043	-0.391372902
